# What Drives Quality Physical Education? A Systematic Review and Meta-Analysis of Learning and Development Effects From Physical Education-Based Interventions

**DOI:** 10.3389/fpsyg.2022.799330

**Published:** 2022-06-23

**Authors:** Dean Dudley, Erin Mackenzie, Penny Van Bergen, John Cairney, Lisa Barnett

**Affiliations:** ^1^Macquarie School of Education, Macquarie University, Sydney, NSW, Australia; ^2^School of Human Movement and Nutrition Science, University of Queensland, Brisbane, QLD, Australia; ^3^The King's School Institute, The King's School, Sydney, NSW, Australia; ^4^Centre for Educational Research, Western Sydney University, Penrith, NSW, Australia; ^5^School of Health and Social Development, Institute for Physical Activity and Nutrition, Deakin University, Geelong, VIC, Australia

**Keywords:** physical education (PE), systematic review and meta-analysis, learning, child development, pedagogy

## Abstract

**Objective:**

To determine the effects of learning interventions aimed at optimizing the quality of physical education (PE) on psychomotor, cognitive, affective and social learning outcomes in children and adolescents.

**Design:**

A systematic review and meta-analysis.

**Data Sources:**

After searching PsycInfo, ERIC, and SportDiscus electronic databases, we identified 135 eligible studies published between January 1, 1995 to May 1, 2021.

**Eligibility Criteria for Selecting Studies:**

We included randomized controlled trials, quasi-experimental studies, and controlled trials that assessed the effect of a PE-based intervention against one of the four identified learning domains in youth at school (aged 5–18 years).

**Results:**

One hundred and thirty five (135) studies with over 42,500 participants and 193 calculated effect sizes were included in the study. The mean effect across all the learning and development outcomes was small to medium (Cohen's *d* = 0.32, 95% confidence interval [CI] (0.27–0.37). When adjusted for publication bias using the Duval and Tweedie Trim and Fill Method, this mean effect size increased to *d* = 0.40 (CI = 0.34–0.46). Effect sizes varied significantly based on learning and development outcomes. Interventions that consistently report above or below the mean *d* = 0.40 effect are identified based on learning outcome. The greatest effects across interventions were witnessed in psychomotor learning outcomes (*d* = 0.52) followed by affective (*d* = 0.47), social (*d* = 0.32), and cognitive (*d* = 0.17) learning outcomes. A minority (<10%) of PE interventions captured by this systematic review and meta-analysis reported having a negative effect on student learning and development.

**Conclusion:**

The interventions with the greatest effects on student learning and development were dependant on the learning domains. Some PE interventions with a pedagogical focus such as games-based approaches, TARGET/Mastery Teaching, and Sport Education were found to be strong investments across multiple domains. The evidence is limited however by consistency in intervention dosage, study design, and data collection instruments. The study received no internal or external funding and was not prospectively registered.

## Introduction

Defining what constitutes “quality physical education” (QPE) has long been an arduous affair within the discipline of physical education (PE), and more broadly in the education, psychology, and public health sciences (Dudley et al., 2020). Indeed, Pate and Hohn (1995) described PE as suffering from a “muddled mission.” Frequently, PE curricula and interventions around the world have been defined to achieve short, medium, or long term health effects including physical activity participation, body adiposity, and other fitness metrics. However, there is now a growing consensus that PE is also a potential mechanism for other aspects of development, with children and young people also needing to learn how to be confident, competent and knowledgeable in order to lead an active life as they age (Cairney et al., 2019; Barnett et al., 2021). Physical activity qua physical activity is not sufficient.

In the first review to consider the role of PE in promoting positive youth development, Bailey (2006) identified the possible benefits of PE curricula and pedagogy that occur across a number of domains: physical, lifestyle, affective, social, and cognitive. He suggested, based on the studies that were qualitatively analyzed, PE has the potential to make significant and distinctive contributions to development in each of these domains. However, the review also stressed that many of these benefits may be mediated by the nature of the instructional interactions between students and their teachers. Thus, the design of the PE lesson and activities is critical.

Nine years later, and after 3 years of consultation, the United Nations Educational, Scientific, and Cultural Organization (UNESCO) released the Quality Physical Education Guidelines for Policymakers (McLennan and Thompson, 2015) which sought to provide further clarity to these claims. The document calls for QPE to drive broad academic achievement and health gains in youth around the world. It also states that growth should occur through fostering an inclusive environment for all students in PE, built upon the philosophical understandings of physical literacy. The inclusion of physical literacy as a central tenet of the QPE aligned with the benefits identified by Bailey (2006) in that it contends for the interconnected and holistic development of youth (Dudley, 2015; Whitehead, 2019). From a learning and pedagogical imperative, physical literacy is best understood as the lifelong learning that occurs across four domains of learning and development (psychomotor, cognitive, affective, and social) and is expressed through individual experiences of movement and physical activity (Keegan et al., 2019).

Recognizing that the psychomotor, cognitive, social, and affective domains are interconnected, and that they represent distinct aspects of a QPE program, it is important that research investigate the evidence for different PE lessons, instructional designs, and interventions which aim to address each domain systematically. In recent years, several systematic reviews and meta-analyses have attempted to make the case for QPE. For instance, one meta-analysis synthesized the effects of physical activity on academic achievement of youth (Alvarez-Bueno et al., 2017), one examined the effects of fundamental movement skill interventions on psychomotor development of youth (Logan et al., 2012), and one reported on the motivations of youth to be physically active (Knittle et al., 2018). Yet, no work to date has examined evidence for learning and development in the psychomotor, cognitive, social, and affective domains simultaneously. Furthermore, whilst Bailey (2006) and UNESCO each suggest that QPE programs should educate children across these domains, no research to date has attempted to capture the teaching and learning strategies that best support this goal.

Another layer of complexity when considering the educational impact of specific PE lessons, instructional designs, and interventions on our four domains of interest is the almost universal positive effect of school-based interventions. As detailed by Hattie (2009) in his seminal synthesis of over 800 meta-analyses on student achievement, almost everything in schools works in some capacity. Ninety percent of all intervention effect sizes are positive. This means that when teachers, schools, and their governing systems claim that they are having a positive effect on student achievement (and, potentially, other developmental outcomes), it becomes almost a trivial claim (Hattie, 2009). While no such research has been conducted in other domains of interest, including for psychomotor outcomes, affective outcomes, social outcomes, or for cognitive outcomes that extend beyond achievement (e.g., executive function, memory, attention), we consider it plausible that PE interventions designed with QPE principles in mind will be similarly beneficial. However, the degree to which they are effective, both between and within domains, remains a question of empirical and practical interest. For this reason, a “hinge point” of average effects across and within learning domains should be predetermined when sufficient evidence is available to determine what programs should receive educational investment and support.

To determine a satisfactory “hinge point,” it is useful to compare intervention effect sizes using a common metric. When standardized, effect sizes allow researchers and policy-makers to compare results on different measures, between groups, over time, and across content of educational interventions (Glass et al., 1981; Hattie, 2009). In Hattie's synthesis of meta-analyses, for example, the average effect of all interventions on student achievement using Cohen's *d*, a measure of the standardized difference between two means, was 0.4 (Hattie, 2009). Thus, in the case of student achievement, 0.4 becomes the relevant hinge-point for the assessment of whether an intervention has a greater or lesser than average impact. While hinge-points may vary across domains, we nonetheless adopt this approach when considering the role of different PE interventions on students' psychomotor, cognitive, affective, and social outcomes.

The aim of this systematic review and meta-analysis was to systematically capture and evaluate the impact of all published PE classes, instructional designs, and interventions on students' psychomotor, cognitive, affective, and social outcomes. Given that QPE must be driven by student outcomes, and drawing on the notion of an effect size “hinge-point” to determine which interventions should receive educational support, this systematic review and meta analyses is the first known study to articulate the PE-based interventions that are having a greater than average effect on cognitive, social, affective, and/or psychomotor learning and development.

## Methods

### Protocol and Registration

To increase the rigor of reporting, we followed the PRISMA 2020 Checklist (Page et al., 2021) as pragmatically possible given this was a systematic review and meta-analysis of predominately educational literature (see Supplementary Material). As our study commenced as a rapid review but evolved into a more rigorous systematic review and meta-analysis, therefore, it was ineligible for registration with the International Prospective Register of Systematic Reviews. Covidence(™) software, as reviewed by Babineau (2014), was used in the title and abstract screening, full-text review, and data extraction phases of the study.

### Eligibility Criteria and Study Selection

To be eligible for inclusion in the review, studies needed to meet the following “PICOS” criteria: (1) **P**articipants: children and adolescents aged 5–18 years old (given prescribed PE usually commences in primary schools) enrolled in school but did not include participants with specific learning needs; (2) **I**ntervention characteristics included studies that used PE classes at school as the intervention medium; (3) **C**omparison: control group that received the regular PE instruction or no PE instruction; (4) **O**utcomes: psychomotor (e.g., gross motor skill, motor competence, fundamental movement skill acquisition), cognitive (e.g., executive function, memory, attention, academic scores); affective (e.g., motivation, self-esteem, self-efficacy, enjoyment, self-regulation), or social (e.g., prosocial behavior, teamwork, cooperation, social competence, self control) outcomes; (5) **S**tudy design: randomized controlled trials (RCTs), quasi experimental trials (QETs), and controlled trials (CTs).

### Search Strategy

Searches of the PsycInfo, ERIC, and SportDiscus databases were conducted. To ensure a comprehensive overview of the field we limited our search to studies published between 1st January 1995 and 1st May 2021. The search was conducted between 1st May to 14th May 2021. Only peer-reviewed journal articles published in English were included. To identify relevant research, we combined three blocks of search terms. Our first group (Block 1) of search terms was used to identify relevant participants. These included variations of “adolescent” OR “child^*^” OR “youth” OR “teen^*^” WITH “primary school” AND/OR “elementary school” OR “middle school” OR “secondary school” OR “high school,” respectively. Our second group of search terms (Block 2) was used to identify research on school-based PE classes. These included the terms “physical education” OR PDHPE OR PESS OR sport OR “health education.” Our final group of search terms (Block 3) was used to identify research that implemented relevant study designs. These included variations of intervention, “randomis(z)ed control trial” OR quasi-experimental OR “control trial” OR “program” OR “comparison.” All three blocked search terms were combined in order for Block 1 AND Block 2 AND Block 3 search terms to be applied concurrently. Given our outcomes of interest include four broad domains, each representing a large range of specific skills and capacities, we did not specify any outcome search terms. We instead used the screening process to identify studies with relevant outcomes falling into each domain.

To complement our database searches, the first author also conducted bi-directional screening of articles using previously published systematic reviews in physical activity, physical literacy, and movement skill development conducted in schools. Bi-directional screening is a method where a reviewer screens all references within an article and any articles that cited the article (Hinde and Spackman, 2015). This process aims to include relevant articles that may have been missed through traditional database searching.

### Data Collection Process

We uploaded all articles captured in the initial database search and complementary bi-directional screening into the Covidence™ review management software library. Using this software, we then removed any duplicates. Two independent reviewers blindly screened titles and abstracts for inclusion or exclusion, with conflicts resolved by consensus by using a third reviewer (the lead author). Articles that were clearly out of scope were excluded, while those with the potential to be in-scope were retained. Next, two independent reviewers independently screened the full-text articles. Only those articles that met the inclusion criteria of the study were retained.

For each study, the following data were extracted: (1) authors' names, year of publication and country of the study; (2) number of participants; (3) characteristics of PE intervention (i.e., pedagogical model, instruction model, policy change); (4) psychomotor, cognitive, affective, and social learning information about instruments used; (5) targeted intervention site (i.e., primary or secondary school); and (6) results in all groups about the parameters of interest. Table 1 provides a summary of the included studies based on learning outcome, study design, country, mode of intervention, and site of intervention. Meta analysis of these data are presented via a series of forest plots based on their level of analysis of the learning domain targeted. A full reference list of studies included in the meta-analysis are provided in the Supplementary Materials attached to this paper.

**Table 1:** An overview of the studies extracted from papers and included in the meta-analysis.

**Author (Year)**	**Country**	**Study design**	**Sample size**	**Primary mode of PE intervention**	**Targeted learning domain(s)**	**Targeted level of schooling**
Abós (2017)	Greece	QE	35	TARGET/Mastery motivational model	Aff, Psy, Soc	Secondary
Aguayo (2019)	Spain	RCT	157	(1) Games pedagogy; (2) Health-based PE	Cog	Primary
Almolda-Tomas (2014)	Spain	CT	113	TARGET/Mastery motivational model	Aff, Psy, Soc	Secondary
Andrade (2020)	Brazil	RCT	140	Exergaming	Aff	Primary
Ardoy (2014)	Spain	RCT	35	Increased PE frequency	Cog	Secondary
Bardaglio (2015)	Italy	CT	128	Team teaching	Psy	Primary
Barkoukis (2008)	Greece	CT	374	TARGET/Mastery motivational model	Aff	Secondary
Barzouka (2015)	Greece	CT	43	Teacher feedback	Psy, Aff	Secondary
Bechter (2019)	Australia	RCT	497	Student-centered	Aff, Psy	Secondary
Benítez-Sillero (2021)	Spain	QE	764	Cooperative games	Soc	Secondary
Bortoli (2015)	Italy	QE	108	TARGET/Mastery motivational model	Aff	Secondary
Boržíková (2020)	Slovakia	RCT	84	Games pedagogy	Psy	Primary
Boyle-Holmes (2010)	USA	QE	1,394	Developmental approach	Aff	Primary
Breslin (2012)	UK	CT	177	Direct instruction	Psy	Primary
Browne (2004)	Australia	CT	53	Sport education	Cog, Psy	Secondary
Carlson (2008)	USA	CT	5,316	Increased PE frequency	Cog	Primary
Cecchini (2007)	Spain	RCT	124	Teaching personal & social responsibility	Aff, Soc	Secondary
Cecchini (2020)	Spain	CT	830	TARGET/Mastery motivational model	Aff	Secondary
Chatoupis (2017)	Greece	RCT	75	(1) Direct instruction; (2) Student-centered	Psy	Primary
Chatzipanteli (2015)	Greece	CT	601	Student based with Mosston Teaching Styles	Cog, Aff	Secondary
Chen (2008)	USA	CT	199	Science-based PE	Aff	Primary
Cheon (2019)	South Korea	RCT	2,739	Autonomy-supported	Aff, Soc, Psy	Secondary
centerCoe (2006)	USA	QE	428	Health-based PE	Cog	Primary
Cohen (2012)	USA	CT	97	Aligned developmental feedback	Psy	Primary
Coimbra (2021)	Switzerland	RCT	143	Goal-setting	Aff	Secondary
Colella (2019)	Italy	RCT	84	Discovery/problem solving approach	Psy, Aff	Primary
Cöster (2018)	Sweden	CT	599	Daily PE	Cog	Primary
Costigan (2016)	Australia	RCT	44	Fitness-based	Cog, Aff	Secondary
Cuevas (2016)	Spain	QE	86	Sport education	Aff	Secondary
Dalziell (2015)	UK	CT	46	Specialist PE	Cog	Primary
Dalziell (2019)	UK	QE	139	Student-centered	Cog, Psy	Primary
De Bruijn (2020)	Netherlands	RCT	654	Increased PE frequency	Cog	Primary
Digelidis (2003)	Greece	CT	783	TARGET/Mastery motivational model	Aff	Secondary
Duncan (2019)	UK	RCT	92	Fitness/neuromuscular	Psy	Primary
Eather (2016)	Australia	RCT	21	Fitness-based	Aff	Secondary
Ellis (1995)	USA	CT	40	Integrated PE	Cog	Primary
Ericsson (2008)	Sweden	CT	152	Increased PE Frequency	Psy, Cog	Primary
Ericsson (2014)	Sweden	CT	220	Increased PE frequency	Psy, Cog	Primary
Escartí (2010)	Spain	CT	42	Teaching personal & social responsibility	Aff, Soc	Primary
Felver (2020)	USA	QE	21	Yoga	Soc	Primary
Fernandez-Rio (2017)	Spain	QE	249	Cooperative Learning	Aff, Soc	Secondary
Fisher (2011)	UK	RCT	57	Health-based PE	Cog	Primary
Font-Lladó (2020)	Spain	RCT	190	Direct instruction	Psy	Primary
Franco (2017)	Spain	QE	53	Self determination theory supported	Aff, Psy	Secondary
Fu (2016)	USA	CT	174	Health-based PE	Aff	Primary
García-Calvo (2016)	Spain	QE	835	(1) Positive behavior model; (2) Didactic	Soc	Secondary
Gibbons (1995)	Canada	RCT	286	(1) Increased PE frequency; (2) Social Learning (Bandura)	Soc	Primary
Gibbons (2010)	Canada	CT	72	Experiential learning	Aff, Soc	Primary/Secondary
Gil-Arias (2017)	Spain	QE	110	(1) Teaching games for understanding; (2) Sport Education	Aff	Secondary
Gråstén (2017)	Finland	CT	240	Constructive alignment	Psy	Secondary
Grasten (2019)	Finland	QE	726	Creative PE	Soc	Primary
Gray (2011)	UK	QE	52	Teaching games for understanding	Psy, Cog	Secondary
Greco (2020)	Italy	CT	100	Health-based PE	Aff	Secondary
Gu (2018)	USA	CT	183	Fitness-based	Psy, Aff	Primary
Hagins (2016)	USA	RCT	104	Yoga	Cog	Secondary
Hartmann (2010)	Switzerland	RCT	231	Daily PE	Soc, Psy	Primary
Harvey (2017)	USA	QE	346	Teaching games for understanding	Aff, Psy, Soc	Primary/Secondary
Hernández (2020)	Spain	QE	102	Autonomy support/dialogic teaching	Psy, Aff, Soc	Primary
Hortz (2008)	USA	QE	240	Health promoting	Soc, Aff	Secondary
How (2013)	Australia	CT	143	Choice-based curriculum	Aff	Secondary
Ignico (2006)	USA	CT	86	Fitness infused	Psy	Primary
Ilker (2013)	Turkey	QE	54	Mastery teaching	Aff	Secondary
Jaakkola (2006)	Turkey	CT	461	TARGET/Mastery motivational model	Aff	Secondary
Jamner (2004)	USA	CT	47	Daily PE	Aff	Secondary
Jansen (2018)	Germany	QE	144	Increased PE frequency	Cog	Secondary
Jarani (2016)	Albania	RCT	1,024	(1) Health-based PE; (2) Games pedagogy	Psy	Primary
Kalaja (2012)	Finland	QE	446	Mastery teaching	Psy	Secondary
Karabourniotis (2002)	Greece	CT	45	Experiential Learning	Psy	Primary
Kliziene (2018)	Lithuania	CT	4028	Psychosocial/Kilaz	Aff	Secondary
Kokkonen (2019)	Finland	CT	382	Creative PE	Aff	Primary
Kouli (2009)	Greece	CT	57	Fitness-based	Aff	Primary
Kriellaars (2019)	Canada	QE	220	Circus arts	Psy	Primary
Krüger (2018)	Germany	QE	61	Sport pedagogy	Cog	Primary
Lakes (2004)	USA	RCT	193	Martial arts	Cog, Soc, Aff	Primary
Lander (2017)	Australia	RCT	190	Constructive alignment	Psy	Secondary
Leptokaridou (2014)	Greece	CT	54	Autonomy supported	Aff	Primary
Lima (2020)	Brazil	RCT	430	Increased PE frequency	Cog	Secondary
lisahunter (2014)	Australia	CT	107	Direct instruction	Cog	Primary
Lonsdale (2019)	Australia	RCT	998	Health-based PE	Aff	Secondary
Lopes (2017)	Portugal	RCT	40	Increased PE frequency	Psy	Primary
Lubans (2018)	Australia	RCT	1,164	Health-based PE	Cog	Secondary
Marshall (1997)	Canada	CT	110	Daily PE	Psy	Primary
Martin (2009)	USA	QE	54	Mastery	Psy	Primary
Martínez-López (2018)	Spain	RCT	184	Fitness-based	Cog	Secondary
Mathisen (2016)	Norway	QE	43	Dynamic systems approach	Psy	Primary
Mayorga-Vega (2012)	Spain	RCT	69	Fitness-based	Aff	Primary
McKenzie (1998)	USA	RCT	508	Health-based PE	Psy	Primary
Meijer (2020)	Netherlands	RCT	1,271	(1) Cognitively challenging; (2) Fitness-based	Cog	Primary
Miller (2016)	Australia	RCT	30	Teaching games for understanding	Cog, Psy, Aff	Primary
Moreno-Murcia (2019)	Spain	CT	20	Task-orientated	Soc, Aff, Psy	Primary
Morgan (2002)	UK/USA	QE	153	TARGET/Mastery motivational model	Aff	Secondary
Neumark-Sztainer (2010)	USA	RCT	336	Health-based PE	Psy, Aff	Secondary
Neville (2021)	UK	CT	40	Dance	Cog	Secondary
Noggle (2012)	USA	RCT	51	Yoga	Aff	Primary
O'Brien (2008)	Ireland	QE	85	Critical theorist PE	Aff	Secondary
Osterlie (2018)	Norway	QE	338	Flipped learning	Aff	Secondary
Pagona (2008)	Greece	CT	60	Metacognitive strategy	Psy	Secondary
Palmer (2018)	USA	CT	260	Meaningful PE	Aff	Primary
Pardo (2016)	Spain	QE	682	Health-based PE	Aff, Psy	Secondary
Perlman (2010)	Australia	QE	78	Sport education	Aff	Secondary
Pesce (2012)	Italy	CT	125	Specialist PE	Soc, Psy	Secondary
Pesce (2016)	Italy	RCT	460	Deliberate play	Psy, Cog	Primary
Pesce (2021)	Italy	RCT	66	Socio-emotional PE	Cog, Soc	Primary
Pietsch (2017)	Germany	CT	46	Cognitive/Motor coordination	Cog	Primary
Platvoet (2016)	Netherlands	CT	244	Goal directed pedagogy	Psy	Primary
Polvi (2000)	Finland	CT	143	(1) Cooperative learning; (2) Direct instruction	Soc	Primary
Potdevin (2018)	France	CT	33	Video feedback	Aff	Primary
Prusak (2004)	USA	RCT	42	Self determination theory supported	Aff	Secondary
Reed (2013)	USA	CT	189	Daily PE	Cog	Primary/Secondary
Robertson (2018)	UK	RCT	136	Exergaming	Aff	Primary
Rubeli (2020)	Switzerland	CT	315	Reflexive pedagogy	Aff	Primary/Secondary
Sallis (1999)	USA	RCT	883	Health-based PE	Cog	Primary
Sánchez-Oliva (2017)	Spain	CT	836	Self determination theory supported	Aff	Secondary
Schmidt (2013)	Switzerland	CT	464	Health-based PE	Aff	Primary
Schmidt (2015)	Switzerland	RCT	124	Games pedagogy	Cog	Primary
Schmidt (2015a)	Switzerland	RCT	90	Cognitively challenging PE	Cog	Primary
Schnider (2021)	Switzerland	RCT	108	Behavioral skill approach	Aff	Secondary
Sgrò (2020)	Italy	QE	77	Teaching games for understanding	Aff, Soc	Secondary
Sharpe (1995)	Canada	CT	55	Cooperative learning	Soc	Primary
Sparks (2017)	Australia	RCT	382	SDT supported	Aff	Secondary
Spittle (2009)	Australia	CT	115	Sport education	Aff, Psy	Secondary
Stojadinović (2020)	Serbia	CT	162	Integrated PE	Psy	Primary
Sun (2012)	USA	RCT	79	Constructivist/ZPD	Cog	Primary
Telford (2012)	Australia	CT	620	Specialist PE	Cog	Primary
van Beurden (2003)	Australia	QE	1,045	Health-based PE	Psy	Primary
van der Fels (2020)	Netherlands	RCT	1,194	1) Fitness-based; (2) Cognitive/Fitness	Psy	Primary
Velez (2010)	USA	RCT	28	Fitness-based	Aff	Secondary
Viciana (2020)	Spain	RCT	109	Sport education	Aff, Psy, Soc	Secondary
Wallhead (2004)	UK	QE	51	Sport education	Aff, Psy	Secondary
Wallhead (2014)	USA	QE	538	Sport education	Aff	Secondary
Weiss (2015)	USA	QE	404	Fitness-based	Aff, Psy, Soc, Cog	Secondary
Yli-Piipari (2018)	USA	RCT	398	Autonomy supported	Aff	Secondary
You (2013)	USA	CT	61	Health-based PE	Aff	Secondary
Zhu (2016)	USA	CT	30	Technology supported PE	Aff	Primary
Zourbanos (2013)	Greece	CT	55	Self talk	Psy	Primary

*RCT, Randomized Controlled Trial; CT, Controlled Trial; QE, Quasi-experimental; ZPD, Zone of Proximal Development; TARGET, Tasks, Authority, Recognition, Grouping, Evaluation, and Time; Aff, Affective Learning Domain; Cog, Cognitive Learning Domain; Psy, Psychomotor Learning Domain; Soc, Social Learning Domain*.

### Quality of Individual Studies

Included articles were assessed for methodological quality using a 10-item quality assessment scale derived from Van Sluijs et al. (2007) (see Table 2). This tool was designed to measure the methodological quality of the effectiveness of interventions to promote physical activity in children and adolescents. For each included article, two reviewers independently assessed whether the assessed item was present or absent. If an item was not described sufficiently it was allocated an absent score. For each article, when 100% agreement did not occur, the lead author reviewed the paper and determined the presence or absence of the item in dispute.

**Table 2:** Methodological quality assessment items (Adapted from Van Sluijs et al., 2007).

**Item**	**Description**
**A**	Key baseline characteristics are presented separately for treatment groups and for randomized controlled trials and controlled trials, positive if baseline outcomes were statistically tested and results of tests were provided.
**B**	Randomization procedure clearly and explicitly described and adequately carried out (generation of allocation sequence, allocation concealment and implementation)
**C**	Validated measures of learning (validation in same age group reported and/or cited)
**D**	Drop out reported and ≤ 20% for < 6-month follow-up or ≤30% for ≥6-month follow-up
**E**	Blinded outcome variable assessments
**F**	Learning assessed a minimum of 6 months after pre-test
**G**	Intention to treat analysis used (participants analyzed in group they were originally allocated to, and participants not excluded from analyses because of non-compliance to treatment or because of some missing data)
**H**	Potential confounders accounted for in outcome analysis (e.g., baseline score, group/cluster, age)
**I**	Summary results for each group + treatment effect (difference between groups) + its precision (e.g., 95% confidence interval)
**J**	Power calculation reported, and the study was adequately powered to detect hypothesized relationships

### Risk of Publication Bias

We conducted two statistical tests to ascertain the degree of publication bias present in the studies. The first was the Classic Fail Safe N (Orwin, 1983) which describes the stability of a significant effect by calculating how many studies with an effect size of zero would need to be added to the meta-analysis before the reported effect lost statistical significance (*p* < 0.05).

The second method was a Trim and Fill (Duval and Tweedie, 2000), which aims both to identify and correct for funnel plot asymmetry that is likely to occur from publication bias. The method involves three steps. The first step is to remove smaller studies causing funnel plot asymmetry. The second step is to deploy the trimmed funnel plot to estimate the true “center” of the funnel. Finally, the third step is to replace the trimmed studies and their missing “equivalents” around the center. As well as providing an estimate of the number of missing studies, an adjusted intervention effect is derived by performing a meta-analysis including the filled studies.

### Data Synthesis and Analysis

All analyses were conducted using Comprehensive Meta Analysis (CMA) software (v3.3; USA). We analyzed effects using the random-effects model according to DerSimonian and Laird (2015). The effect sizes were expressed as a standardized effect size (Cohen's *d*) for comparison to broader educational research (Cohen et al., 2017).

It is important to clarify the following statistical aspects: (1) when two or more intervention groups using different strategies were included in a study, their data were analyzed as independent studies; (2) when a paper reported testing for more than one learning domain outcome, the data were analyzed as separate studies at the domain level only; (3) when two or more tests for measuring the same learning outcomes variable were included in the same study, they were calculated as a combined standardized effect size (Borenstein, 2021); (4) a random effects model was also applied to compare effect size differences between PE interventions based on learning outcome (i.e., cognitive, social, psychomotor, affective, or combined PE interventions) and the pedagogical (i.e., model of practice, teaching strategy) or intervention approach (i.e., teacher training, policy changes); (5) when two or more age cohorts were included in studies, their data were investigated as combined samples; and (6) when two or more follow-up measurements were reported, only the last measurement was considered.

Heterogeneity was assessed and reported across all the studies and at the learning domain level by using a series of complimentary statistical analyses. First, we calculated the Q-statistic (Q) which provided a test of the null hypothesis as to whether all studies in the model shared a common effect size. Second, we calculated an inconsistency index (I^2^) statistic in order to report the proportion of the observed variance that were indicative of changes in true effect sizes rather than sampling error. Third, a Tau statistic (T^2^) was calculated to determine the variance of true effect sizes; and finally, we calculated and reported a prediction interval to provide a range of true effect size for all samples observed within 95% confidence limits.

## Results

### Study Selection

**Figure 1:**
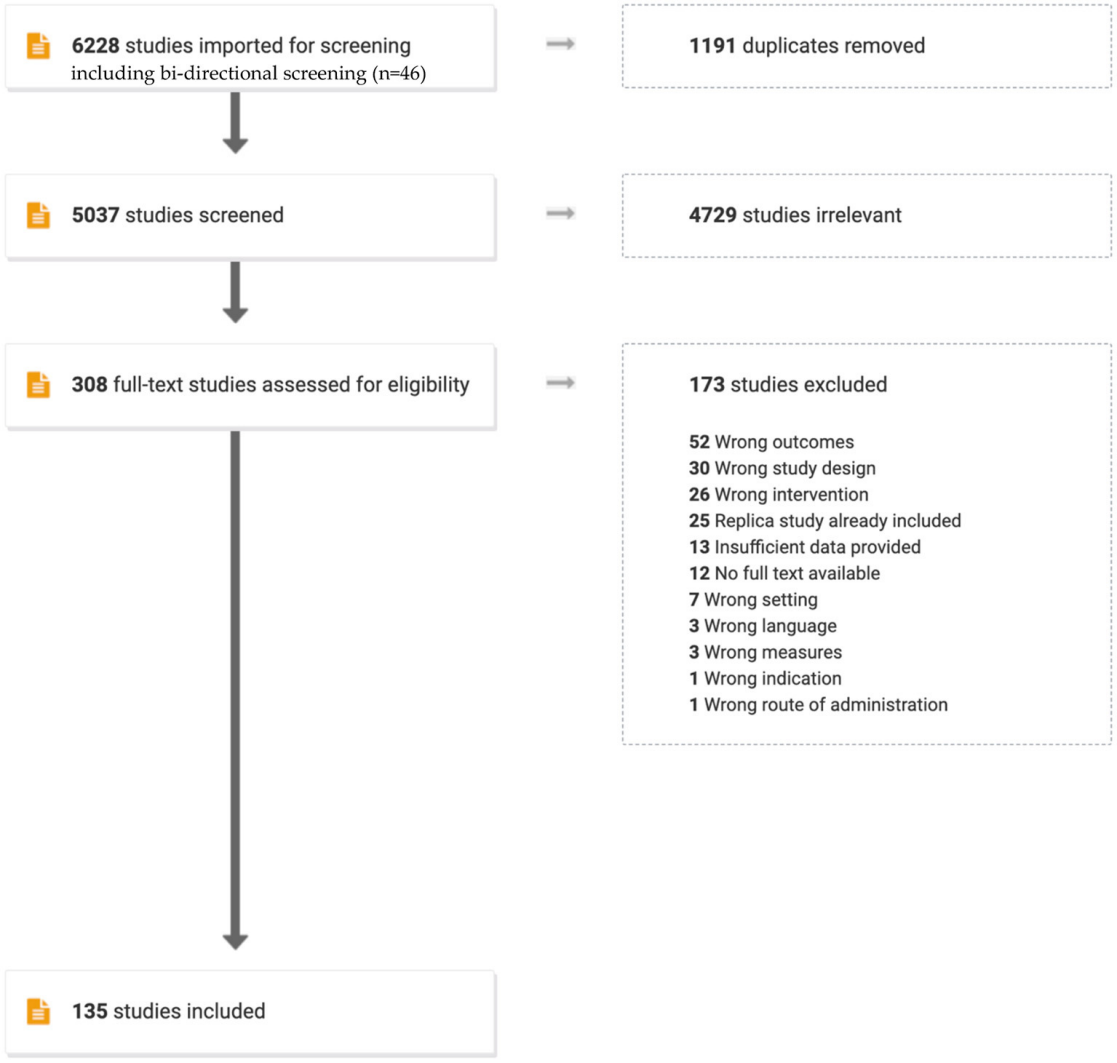
Flowchart of study selection.

The database search strategy yielded 6,182 studies for possible inclusion. The bidirectional screening added an additional 46 studies, resulting in 6,228 studies that were imported for screening. After duplicates were removed by Covidence™, 5,037 papers were reviewed by title and abstract. 4,729 were deemed irrelevant by two reviewers based on their title and abstract resulting in 308 papers being subjected to a full-text review. At this stage, an additional 173 studies were excluded by consensus (as previously discussed) and their reasons for exclusion are detailed in the flowchart in Figure 1.

### Study Characteristics

There were 135 studies extracted from the search strategy for final analysis (see Table 1). Studies came from primary (*n* = 60), secondary (*n* = 71) and a combination of primary and secondary (*n* = 4) school. They consisted of 47 randomized controlled trials, 54 controlled trials, and 34 quasi-experimental studies. The studies captured by the systematic review came from 23 different countries and included over 42,500 child and adolescent participants.

### Risk of Bias Based on Quality of Individual Studies

The evaluation of methodological quality reported in Table 3 revealed that the 135 studies included in the analysis varied considerably in their reporting. Approximately half (56%) of the papers met five or more of the quality assessment criteria (Van Sluijs et al., 2007). Only 16% (*n* = 21) of the collected studies met seven or more assessment criteria. The most infrequently reported quality assessment criteria reported in these studies were blinded assessments (9%; *n* = 12) and appropriate power calculations (16%; *n* = 22), and the most frequently reported were a summary of key findings and the reporting of validated instruments (94%, *n* = 127; 81%, *n* = 110), respectively.

**Table 3:** Results of methodological quality assessment.

**Paper No**	**Paper lead author (Year)**	**Methodological quality assessment items**										**No. of criteria met**
		**A**	**B**	**C**	**D**	**E**	**F**	**G**	**H**	**I**	**J**	
1	Abós (2017)	✓		✓	✓					✓		4
2	Aguayo (2019)	✓	✓	✓						✓		4
3	Almonda-Tomas (2014)			✓				✓		✓		3
4	Andrade (2020)	✓	✓	✓					✓	✓	✓	6
5	Ardoy (2014)	✓	✓	✓		✓			✓	✓		6
6	Bardaglio (2015)	✓								✓		2
7	Barkoukis (2008)			✓			✓			✓		3
8	Barzouka (2015)	✓		✓						✓		3
9	Bechter (2019)	✓	✓	✓	✓	✓				✓	✓	7
10	Benitez-Sillero (2021)	✓		✓						✓		3
11	Bortoli (2015)	✓		✓	✓					✓		4
12	Borzikova (2020)	✓					✓			✓		3
13	Boyle-Holmes (2010)				✓		✓	✓	✓	✓		5
14	Breslin (2012)	✓		✓		✓	✓		✓	✓		6
15	Browne (2004)	✓								✓		2
16	Carlson (2008)	✓	✓	✓			✓		✓	✓		6
17	Cecchini (2007)	✓	✓	✓	✓					✓		5
18	Cecchini (2020)	✓		✓			✓	✓	✓	✓		6
19	Chatoupis (2017)	✓							✓	✓		3
20	Chatzipanteli (2015)	✓	✓	✓						✓		4
21	Chen (2008)	✓	✓	✓			✓	✓		✓		6
22	Cheon (2019)	✓	✓	✓	✓		✓	✓	✓	✓		8
23	Coe (2006)	✓	✓		✓		✓		✓	✓		6
24	Cohen (2012)	✓		✓						✓		3
25	Coimbra (2021)	✓	✓	✓	✓				✓	✓	✓	7
26	Colella (2019)	✓								✓		2
27	Coster (2018)	✓		✓			✓			✓		4
28	Costigan (2016)	✓	✓	✓	✓	✓		✓	✓	✓	✓	9
29	Cuevas (2016)	✓		✓						✓		3
30	Dalziell (2015)			✓						✓		2
31	Dalziell (2019)			✓	✓		✓			✓	✓	5
32	De Bruijn (2020)	✓	✓	✓	✓	✓			✓	✓	✓	8
33	Digelidis (2003)	✓		✓			✓			✓		4
34	Duncan (2019)	✓	✓	✓	✓				✓	✓	✓	7
35	Eather (2016)	✓	✓	✓	✓	✓			✓	✓	✓	8
36	Ellis (1995)									✓		1
37	Ericsson (2008)			✓			✓					2
38	Ericsson (2014)	✓		✓			✓			✓		4
39	Escarti (2010)			✓			✓			✓		3
40	Felver (2020)	✓		✓	✓					✓		4
41	Fernandez (2017)	✓		✓						✓		3
42	Fisher (2011)	✓	✓	✓	✓	✓			✓	✓		7
43	Font-Llado (2020)	✓	✓	✓	✓	✓			✓	✓		7
44	Franco (2017)	✓		✓	✓					✓		4
45	Fu (2016)	✓		✓	✓				✓	✓		5
46	Garcia-Calvo (2016)	✓		✓	✓				✓	✓		5
47	Gibbons (1995)		✓	✓	✓		✓			✓		5
48	Gibbons (2010)	✓		✓			✓		✓	✓		5
49	Gil-Arias (2017)	✓		✓						✓		3
50	Grasten (2017)	✓		✓			✓		✓	✓		5
51	Grasten (2019)			✓				✓		✓		3
52	Gray (2011)	✓								✓		2
53	Greco (2020)	✓		✓	✓			✓		✓	✓	6
54	Gu (2018)	✓	✓	✓	✓				✓	✓		6
55	Hagins (2016)	✓	✓	✓	✓		✓	✓	✓	✓		8
56	Hartmann (2010)	✓	✓	✓	✓		✓		✓	✓		7
57	Harvey (2017)			✓					✓	✓		3
58	Hernandez (2020)	✓		✓			✓			✓		4
59	Hortz (2008)	✓		✓	✓				✓	✓	✓	6
60	How (2013)							✓	✓	✓		3
61	Ignico (2006)						✓		✓			2
62	Ilker (2013)			✓	✓				✓	✓		4
63	Jaakkola (2006)			✓	✓		✓		✓	✓		5
64	Jamner (2004)			✓	✓				✓	✓		4
65	Jansen (2018)	✓		✓					✓	✓	✓	5
66	Jarani (2015)	✓	✓		✓				✓	✓		5
67	Kalaja (2012)	✓		✓	✓		✓			✓		6
68	Karabourniotis (2002)			✓					✓			2
69	Kiliziene (2018)	✓	✓	✓	✓		✓			✓		6
70	Kokkonen (2019)	✓		✓	✓		✓		✓	✓		6
71	Kouli (2009)								✓	✓		2
72	Kriellaars (2019)			✓	✓		✓		✓	✓	✓	6
73	Kruger (2018)	✓		✓	✓			✓	✓	✓		6
74	Lakes (2004)	✓		✓	✓				✓	✓		5
75	Lander (2017)	✓	✓	✓	✓				✓	✓		6
76	Leptokaridou (2016)	✓		✓	✓			✓	✓	✓		6
77	Lima (2020)	✓	✓		✓		✓		✓	✓		6
78	lisahunter (2014)	✓		✓					✓	✓		4
79	Lonsdale (2019)	✓	✓	✓	✓	✓	✓	✓	✓	✓	✓	10
80	Lopes (2017)	✓			✓		✓	✓	✓	✓		6
81	Lubans (2018)	✓	✓	✓	✓	✓	✓		✓	✓	✓	9
82	Marshall (1997)			✓	✓				✓	✓		4
83	Martin (2009)	✓		✓					✓			3
84	Martinez-Lopez (2018)	✓	✓	✓	✓	✓			✓	✓		7
85	Mathisen (2016)	✓								✓		2
86	Mayorga-Vega (2012)		✓	✓	✓					✓		4
87	McKenzie (1998)	✓			✓		✓		✓	✓		5
88	Meijer (2021)	✓	✓	✓	✓				✓	✓	✓	7
89	Miller (2016)	✓	✓	✓	✓			✓	✓	✓	✓	8
90	Moreno-Murcia (2019)	✓		✓	✓					✓		4
91	Morgan (2002)			✓						✓		2
92	Neumark-Sztainer (2010)	✓		✓	✓		✓		✓	✓		6
93	Neville (2021)	✓		✓	✓			✓	✓	✓		6
94	Noggle (2012)	✓	✓	✓	✓			✓	✓	✓		7
95	O'Brien (2008)	✓		✓	✓				✓	✓		5
96	Osterlie (2018)	✓	✓	✓	✓					✓		5
97	Pagona (2008)	✓			✓		✓	✓		✓		5
98	Palmer (2018)	✓	✓	✓	✓				✓			5
99	Pardo (2016)	✓		✓	✓		✓		✓	✓		6
100	Perlman (2010)	✓	✓	✓				✓		✓		5
101	Pesce (2012)				✓		✓		✓	✓		4
102	Pesce (2016)	✓	✓	✓	✓			✓	✓	✓	✓	8
103	Pesce (2020)	✓	✓	✓	✓		✓		✓	✓		7
104	Pietsch (2017)	✓	✓	✓					✓	✓		5
105	Platvoet (2016)			✓					✓	✓		3
106	Polvi (2000)			✓	✓		✓			✓		4
107	Potdevin (2018)				✓					✓		2
108	Prusak (2004)		✓	✓						✓		3
109	Reed (2013)			✓			✓		✓	✓		4
110	Robertson (2018)		✓	✓					✓	✓	✓	5
111	Rubeli (2020)	✓		✓			✓	✓	✓	✓		6
112	Sallis (1999)	✓	✓	✓			✓		✓	✓		6
113	Sánchez-Oliva (2017)	✓		✓	✓				✓	✓		5
114	Schmidt (2013)	✓		✓	✓			✓	✓	✓		6
115	Schmidt (2015)	✓	✓	✓	✓				✓	✓		6
116	Schmidt (2015a)	✓	✓	✓	✓			✓	✓	✓	✓	8
117	Schnider (2021)	✓	✓						✓	✓	✓	5
118	Sgro (2020)			✓	✓					✓		3
119	Sharpe (1995)						✓					1
120	Sparks (2017)	✓	✓	✓	✓	✓		✓	✓	✓	✓	9
121	Spittle (2009)			✓					✓	✓		3
122	Stodjadinovic (2020)	✓			✓				✓	✓		4
123	Sun (2012)		✓	✓	✓			✓	✓	✓		6
124	Telford (2012)	✓	✓	✓	✓		✓		✓			6
125	van Beurden (2003)		✓	✓			✓	✓	✓			5
126	van der Fels (2020)	✓	✓	✓					✓	✓	✓	6
127	Velez (2010)			✓	✓					✓		3
128	Viciana (2020)	✓		✓	✓				✓	✓		5
129	Wallhead (2004)	✓	✓	✓	✓		✓		✓	✓		7
130	Wallhead (2014)			✓	✓		✓			✓		4
131	Weiss (2015)			✓			✓		✓	✓		4
132	Yli-Piipari (2018)	✓		✓					✓			3
133	You (2013)			✓				✓		✓		3
134	Zhu (2016)							✓		✓		2
135	Zourbanos (2013)							✓	✓	✓		3
**% of criteria present**		70%	37%	81%	54%	9%	35%	21%	61%	94%	16%	

### Summary of Evidence

From the 135 included studies, there were 193 effect sizes that examined a learning outcome or outcomes within at least one of the four learning domains of interest (cognitive, social, affective, and/or psychomotor). There were 57 different intervention strategies identified across the studies. Each of these intervention strategies are reported in forest-plots.

#### Combined Learning Effects

As above, all analyses were based on PE interventions targeting a learning variable of interest among students who were aged 5–18 years of age and attending primary and/or secondary schools. In each study, students were assigned to either (i) a new PE program or learning intervention (intervention condition), or (ii) their regular curricula (control condition). Researchers recorded student outcomes at either the conclusion of the intervention period or a later follow-up time point. The effect size was the standardized mean difference (Cohen's *d*) in each outcome variable between intervention and control groups.

The studies in this analysis were sampled from a universe of possible studies defined by the inclusion/exclusion criteria defined earlier in the paper. For this reason, a random-effects model was employed. The conclusion (below) applies to that universe.

##### Do Physical Education Interventions Improve Combined Student Outcomes?

When combined across domains, the PE interventions included in this meta-analysis had a significant positive effect on student outcomes across domains, *Z* = 12.974, *p* < 0.001. The standardized difference in means was *d* = 0.32 (95% CI [0.27, 0.37]), meaning that on average, students receiving a PE-based intervention improved their learning or development by a third of a standard deviation compared with those students who received their usual PE. Primary/elementary school interventions reported a slightly smaller average effect size of *d* = 0.29, whilst interventions conducted in secondary schools were slightly higher at *d* = 0.34.

##### How Much Does the Effect Size Vary Across Studies (Heterogeneity)?

The Q-value is 2481.377 with 134 degrees of freedom and *p* < 0.001. Thus, we acknowledge that the true effect size is not identical in all studies. The *I*^2^ is 94.600% representing the proportion of the observed variance that was indicative of changes in true effect sizes rather than sampling error. The T^2^ is 0.069 representing the variance of true effect sizes and T is 0.262. The prediction interval is −0.198 to 0.846 therefore we would expect the true effect size for 95% of all students receiving the interventions to fall within this range.

##### To What Extent Would Publication Bias or the Small-Study Effect Alter These Findings?

The Classic fail-safe analysis showed that the incorporated data from the 135 observed studies yielded a z-value of 34.888 and corresponding 2-tailed *p* < 0.0001. The fail-safe N suggests that 12,641 “null” effects would need to be included for a combined 2-tailed *p* > 0.05 (i.e., for the reported effect to be nullified).

**Figure 2:**
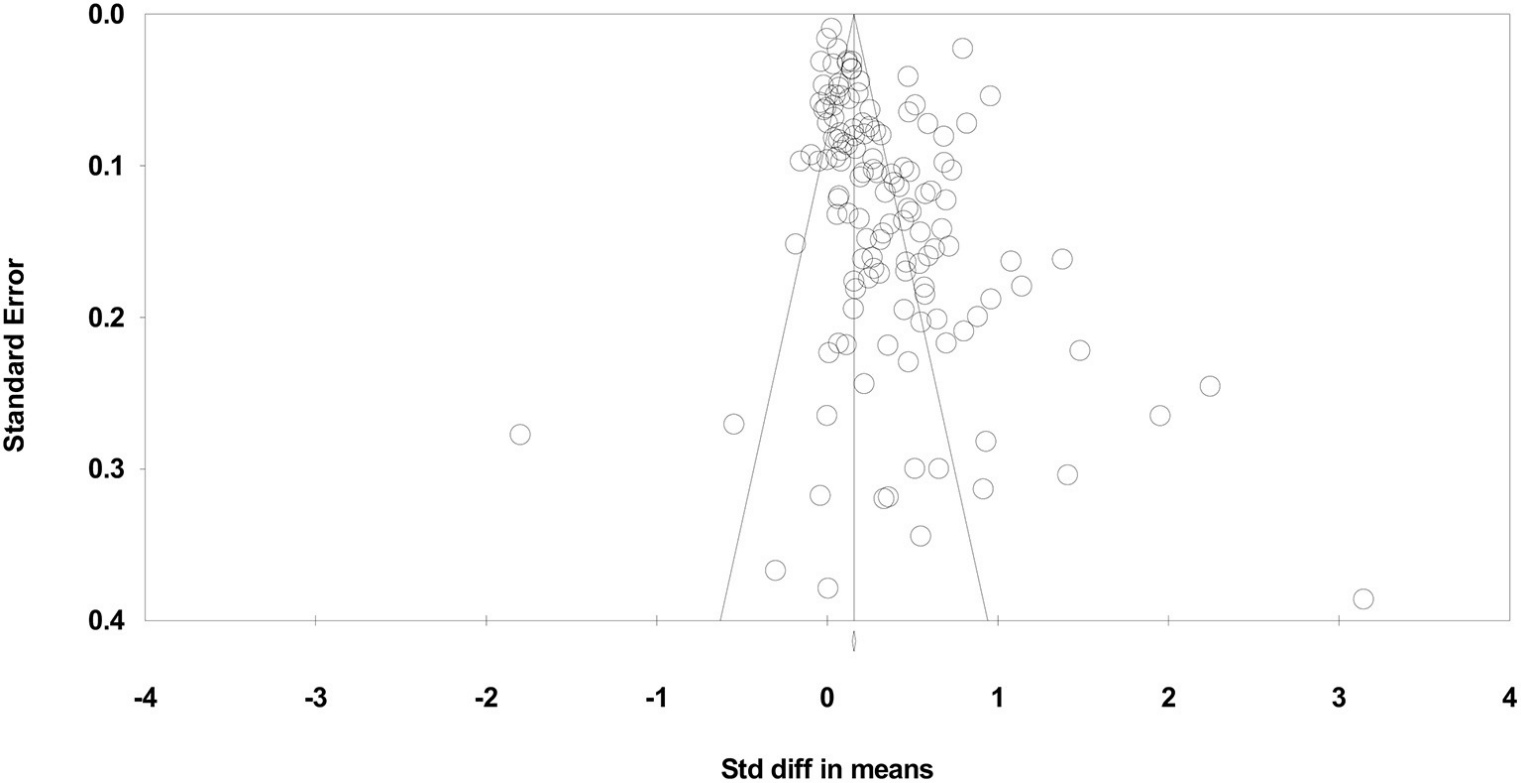
Funnel plot of standard error by standardized difference in mean of combined learning effects.

Duval and Tweedie (2000) “Trim and Fill” results suggest that 18 small studies should be trimmed from the right of the mean to determine the true “center” of the funnel plot and replace them with missing equivalents around the center. The adjusted standardized effect size of the included PE interventions was increased from *d* = 0.32 to *d* = 0.40 (see Figure 2).

#### Cognitive Learning and Development

There were 37 studies employing 39 PE intervention strategies that sought to improve cognitive learning or development, therefore 39 effect sizes are captured in this meta-analysis. Of these 37 studies, 68% (*n* = 25) of the papers met five or more of the quality assessment criteria.

##### Which Physical Education Interventions Most Affect Students' Cognitive Learning and Development?

**Figure 3:**
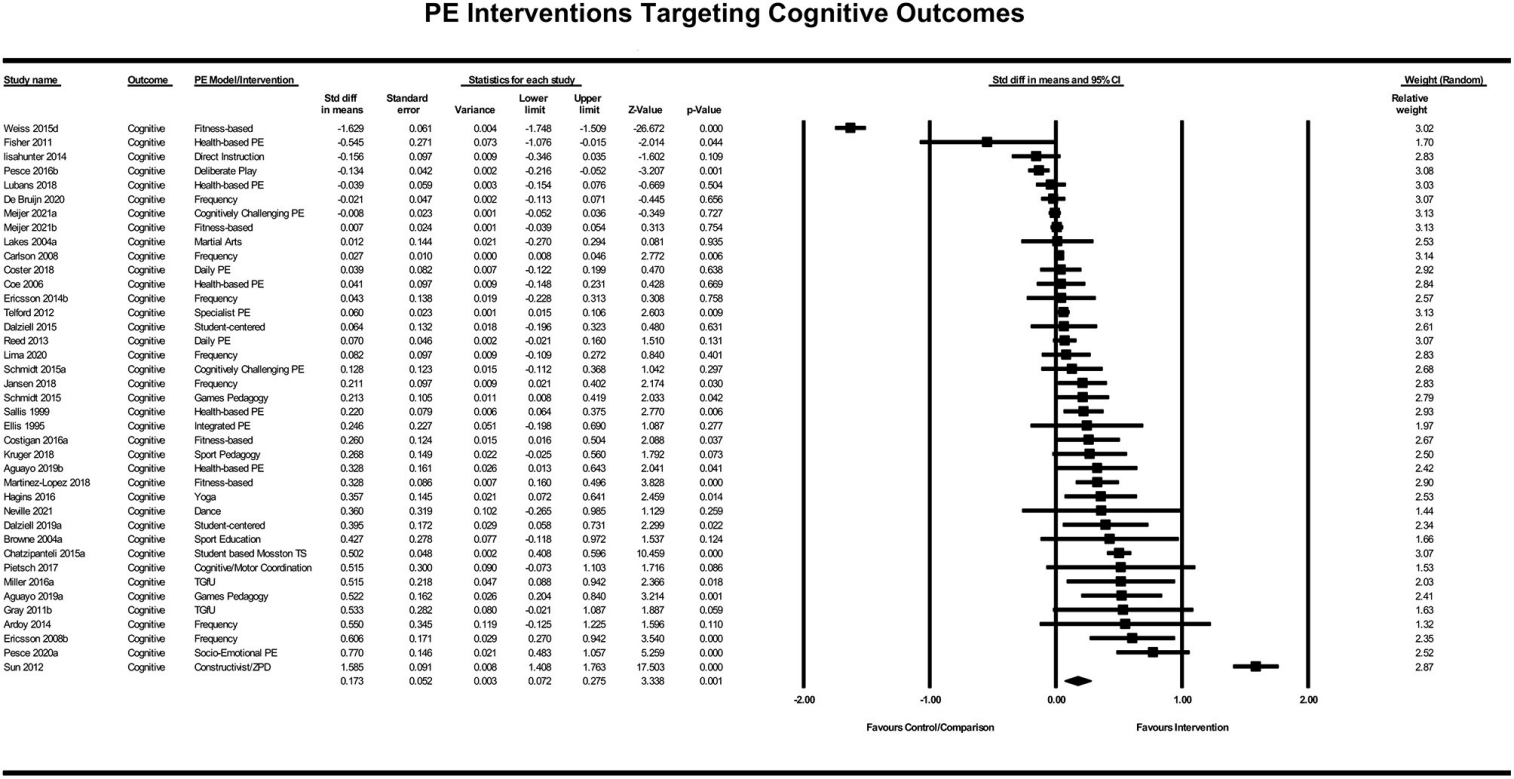
Forrest plot of standardized difference in means of cognitive PE interventions.

The included physical education interventions had a significant positive effect on cognitive outcomes, *Z* = 3.338, *p* < 0.001. The standardized difference in means for all cognitive interventions was *d* = 0.17 (95% CI [0.072, 0.275]; see Figure 3), meaning that on average, students receiving an intervention improved their cognitive learning or development by nearly a fifth of a standard deviation compared with those students who did not receive the same intervention.

Only one PE intervention strategy (represented by three or more studies) had an average effect size on cognitive learning and development that was higher than the domain average of *d* = 0.17. This strategy was to adopt games-based approaches (4 studies; *d* = 0.38), which included the Teaching Games for Understanding model (Bunker and Thorpe, 1983). Most strategies that were represented by three or more studies fell below the domain average of *d* = 0.17. These included (a) increased PE frequency (7 studies; *d* = 0.10); (b) health-based/PA promoting PE (5 studies; *d* = 0.06) and; (c) fitness-based PE (4 studies; *d* = −0.26).

##### Heterogeneity of Cognitive Effects

The Q-value for effects in the cognitive domain was 1269.249 with 38 degrees of freedom and *p*<0.001. The *I*^2^ is 97.006%, T^2^ is 0.086 and T is 0.293. The prediction interval is −0.4310 to 0.7770 and therefore expect the true effect size for 95% of all students receiving a PE intervention to improve cognitive learning to fall within this range.

##### To What Extent Would Publication Bias Alter These Findings?

**Figure 4:**
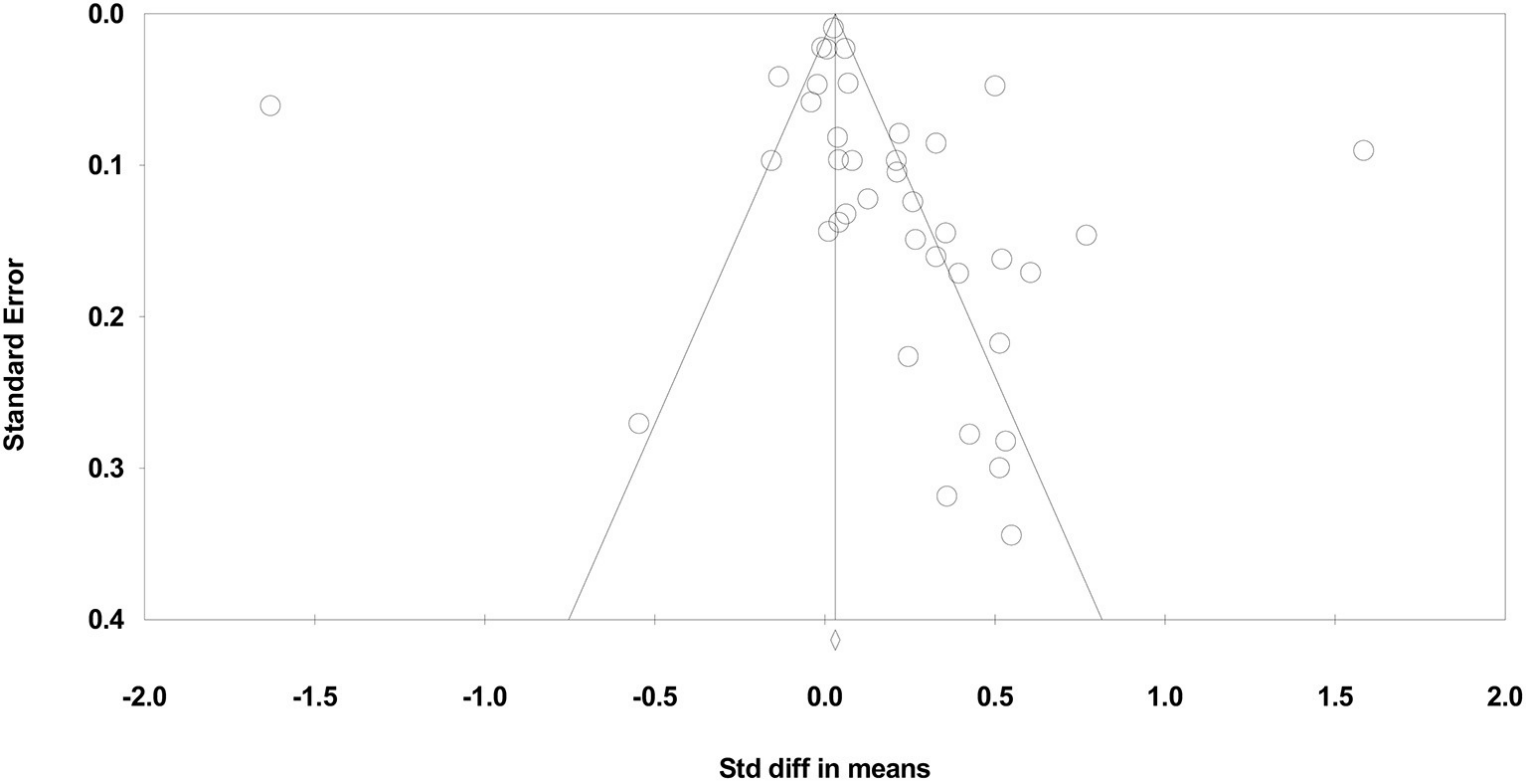
Funnel plot of standard error by standardized difference in means of cognitive PE interventions.

The results of the Classic fail-safe analysis showed that the incorporated data from 39 effects yielded a *z*-value of 7.793 and corresponding 2-tailed *p* < 0.0001. The fail-safe N in this case is 578 suggesting that this many “null” effects would need to be included for a combined 2-tailed *p* > 0.05 (i.e., for the effect to be nullified). According to the “Trim and Fill” analysis, 15 effects could be trimmed from the left of the mean to reduce the potential publication bias. The adjusted standardized effect size for PE interventions on cognitive learning in this case would be decreased to *d*=-0.02 (see Figure 4).

#### Social Learning and Development

There were 25 studies with 29 PE intervention strategies that examined social learning and development in PE. Of these 25 studies, 40% (*n* = 10) met five or more of the quality assessment criteria outlined by Van Sluijs et al. (2007).

##### Which Physical Education Interventions Most Affect Students' Social Outcomes?

**Figure 5:**
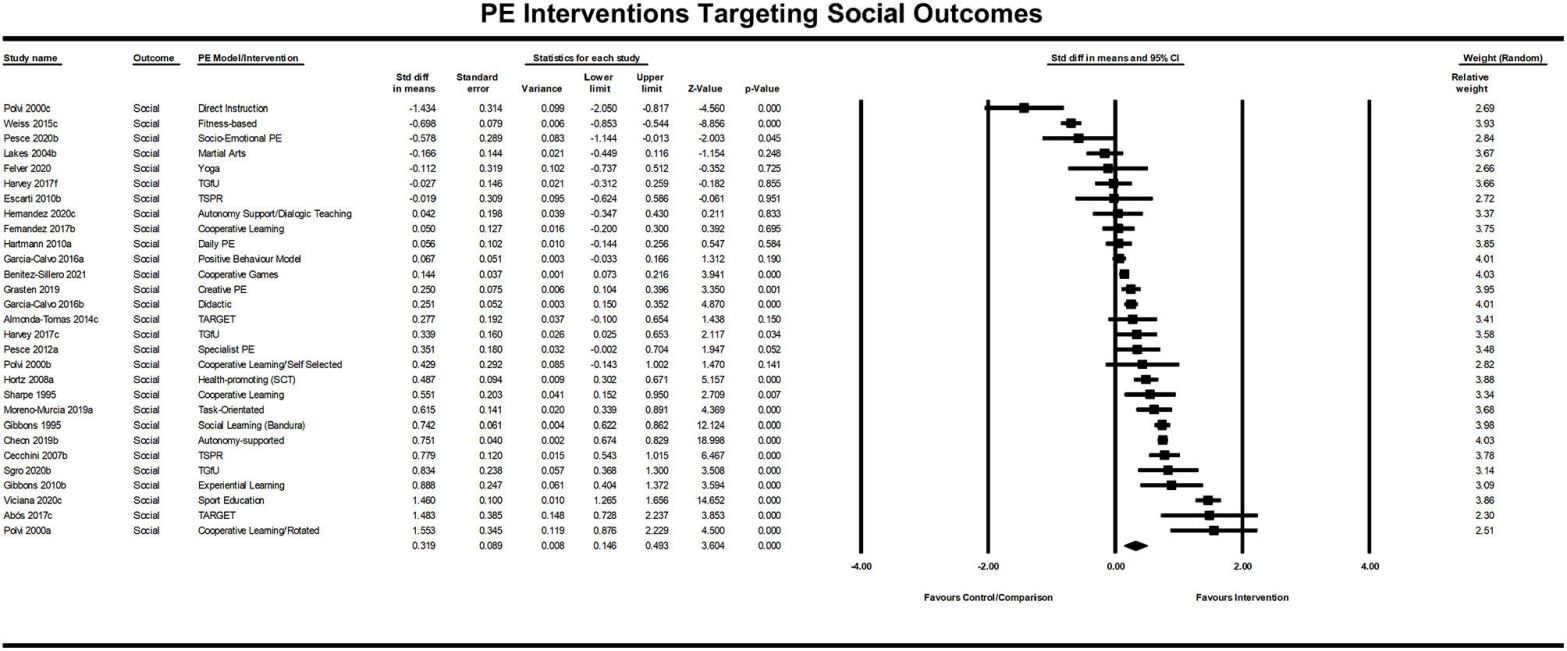
Forrest plot of standardized difference in means of social PE interventions.

The included PE interventions had a significant positive effect on social learning and development, *Z* = 3.604, *p* < 0.001. The standardized difference in means was *d* = 0.32 (95% CI [0.146, 0.493]; (see Figure 5), meaning that students receiving a new instructional design or intervention improved their social learning or development by just under a third of a standard deviation compared with those students who did not receive the intervention.

The PE intervention pedagogy (with three or more studies) that yielded a combined average effect above the domain average of *d*=0.32 were the cooperative learning strategies (4 studies; *d* = 0.42). There were an insufficient number of studies employing a similar intervention strategy to analyse those that are likely to consistently fall below the hinge point for social learning and development.

##### Heterogeneity of Social Effects

The Q-value for these effects is 643.085 with 28 degrees of freedom and *p* < 0.001 (*I*^2^ = 95.646%; *T*^2^ = 0.193; *T* = 0.439). The prediction interval is −0.6006 to 1.2386. We would expect the true effect size for 95% of all students receiving a social learning PE intervention to fall within this range.

##### To What Extent Would Publication Bias Alter These Findings?

**Figure 6:**
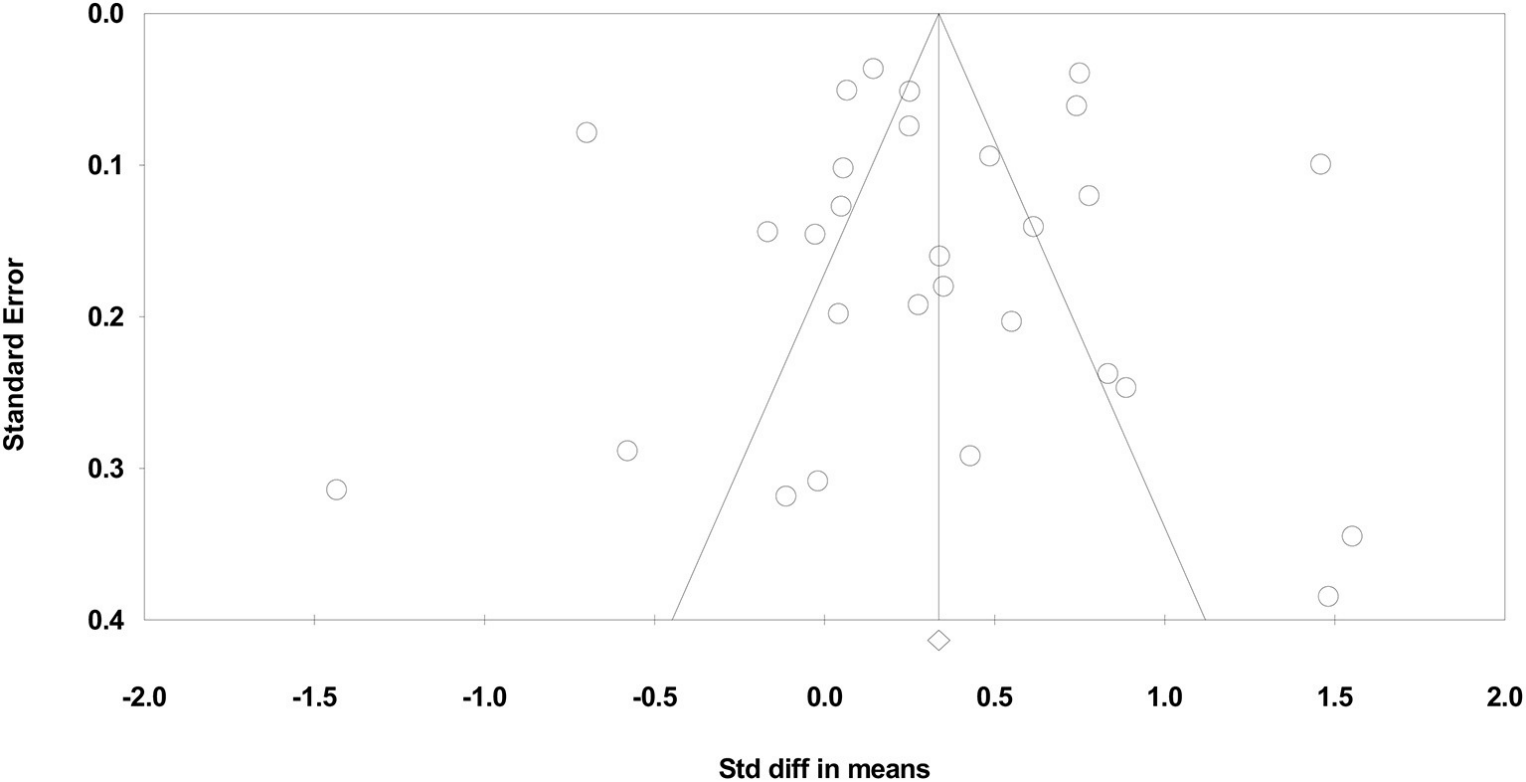
Funnel plot of standard error by standardized difference in means of social PE interventions.

The results of the Classic fail-safe analysis showed that the incorporated data from 29 effects yielded a z-value of 15.665 and corresponding 2-tailed *p* < 0.0001. The fail-safe N in this case is 1,824. The “Trim and Fill” analysis indicates no studies should be trimmed from the left or right of the mean to reduce the potential publication bias (see Figure 6).

#### Psychomotor Learning and Development

There were 51 studies with 55 independent or combined effect sizes that examined psychomotor learning and development. 45% (*n* = 23) of these 51 papers met five or more of the quality assessment criteria.

##### Which Physical Education Interventions Most Affect Student Psychomotor Learning and Development?

**Figure 7:**
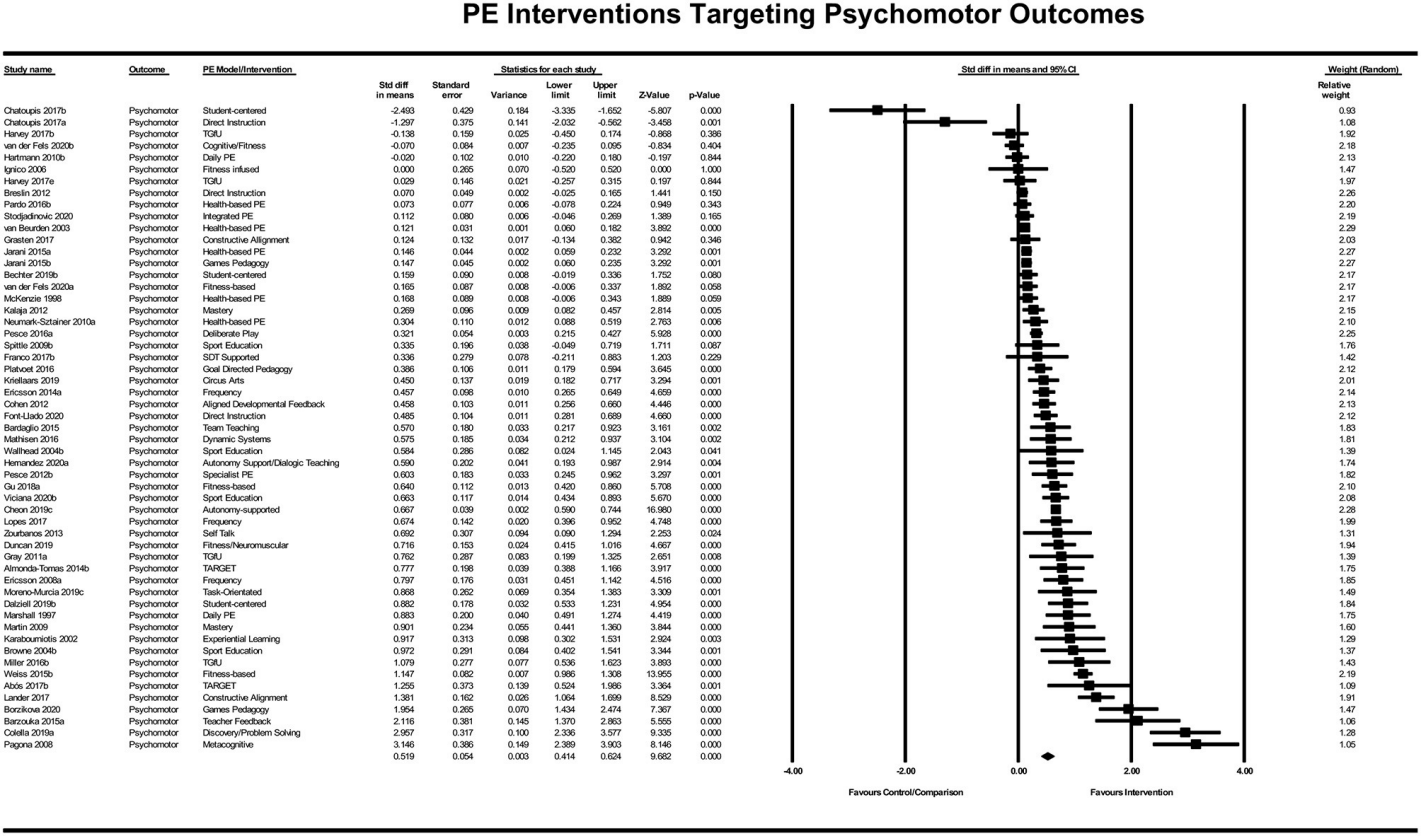
Forrest plot of standardized difference in means of psychomotor PE interventions.

The included PE interventions had a significant positive effect on psychomotor outcomes, *Z* = 9.682, *p* < 0.001. The standardized difference in means was *d* = 0.52 (95% CI [0.414, 0.624]), meaning that students receiving the intervention improved their psychomotor learning and development by just over a half of a standard deviation on average relative to those students who did not receive the intervention (see Figure 7).

The PE intervention strategies (represented by three or more studies) that yielded a combined average effect size on psychomotor learning or development above the domain average of *d* = 0.52 were (a) fitness-based/infused PE models (5 studies; *d* = 0.56); (b) Games-based and Teaching Games for Understanding approaches (5 studies; *d* = 0.58); (c) Mastery and TARGET (Epstein, 1987) PE models (4 studies; *d* = 0.73); (d) Sport Education (Siedentop, 1998) Model (4 studies; *d* = 0.61); and (e) increased frequency of PE (3 studies; *d* = 0.61). Interventions with three or more studies that had an average effect size below the domain-average “hinge point” were the health-based/PA promoting interventions (5 studies; *d* = 0.13).

##### Heterogeneity of Psychomotor Effects

The Q-value for these effects was 753.063 with 54 degrees of freedom and *p* < 0.001 (*I*^2^ = 92.829%; *T*^2^ = 0.124; *T* = 0.353). The prediction interval is −0.1954 to 1.2334 with the true effect size for 95% of all students receiving the interventions to fall within this range.

##### To What Extent Would Publication Bias Alter These Findings?

**Figure 8:**
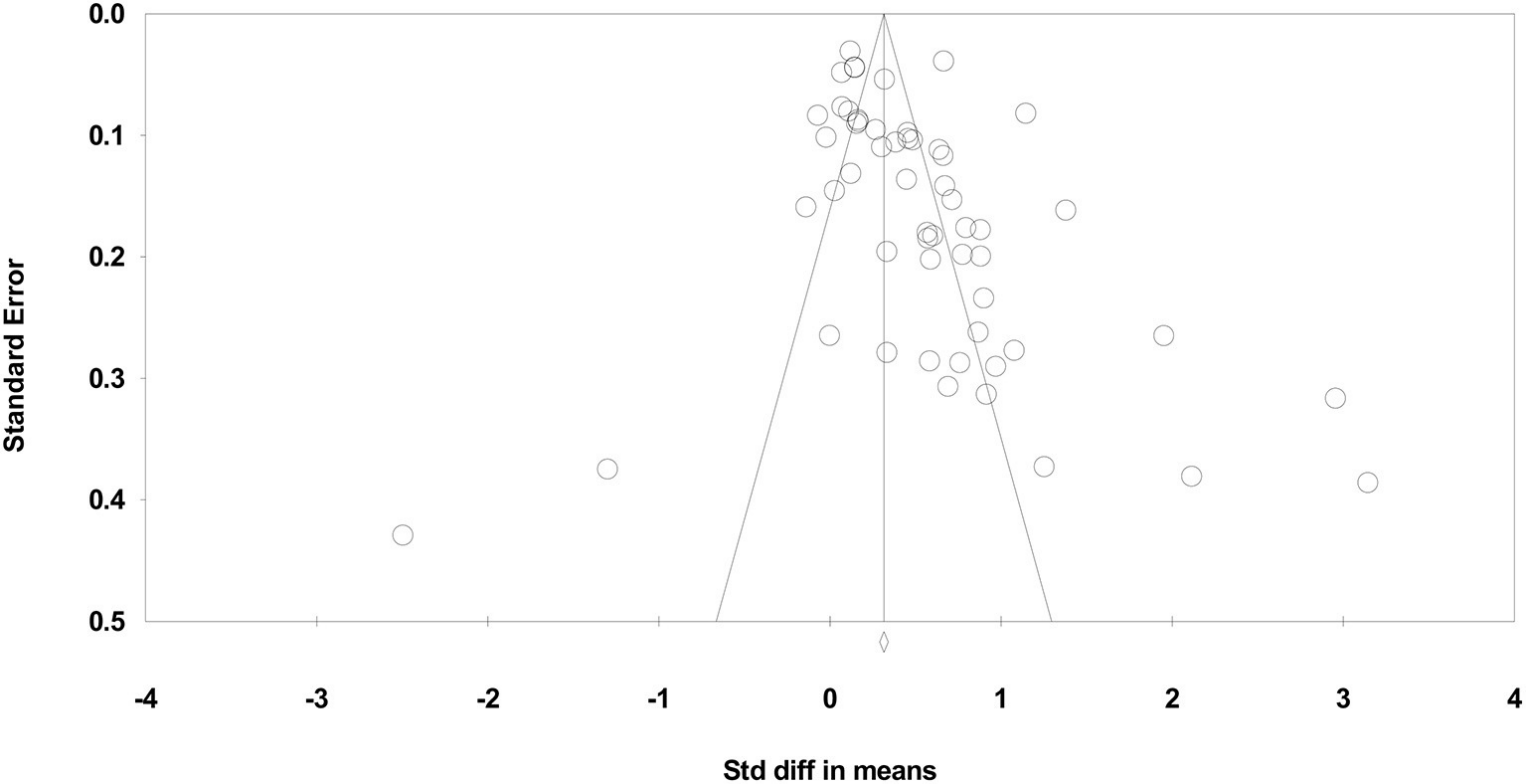
Funnel plot of standard error by standardized difference in means of psychomotor PE interventions.

The results of the Classic fail-safe analysis showed that the incorporated data from 54 effects yielded a *z*-value of 26.085 and corresponding 2-tailed *p* < 0.0001. The fail-safe N in this case is 9688. The “Trim and Fill” analysis indicates no studies should be trimmed in the random effects model from either side of the mean to reduce the potential publication bias (see Figure 8).

#### Affective Learning and Development

There were 69 studies with 71 calculated effect sizes that examined affective learning. 54% (*n* = 37) of these papers met five or more of the methodological quality assessment criteria.

##### Which Physical Education Interventions Most Affect Student Affective Learning?

**Figure 9:**
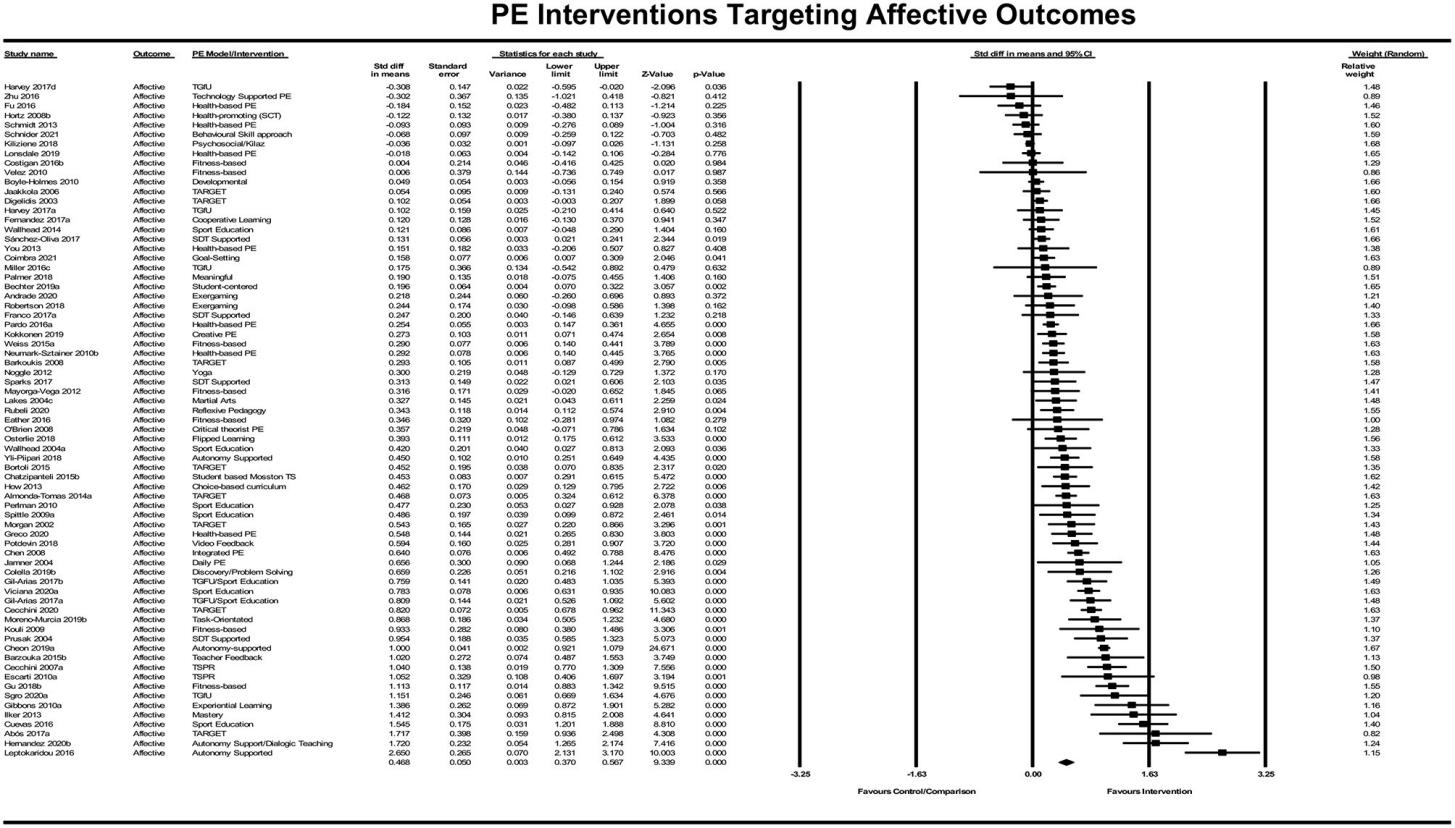
Forrest plot of standardized difference in means of affective PE interventions.

The included PE interventions had a significant positive effect on affective learning, *Z* = 9.339, *p* < 0.001. The standardized difference in means for all affective learning interventions was *d* = 0.47 (95% CI [0.370, 0.567]; see Figure 9), meaning that on average, students receiving the intervention improved their affective learning by just under a half of a standard deviation compared with those students who did not receive the intervention.

There were multiple PE intervention strategies focusing on affective learning that were represented by three or more studies and yielded a combined average effect size above the standardized mean effect for affective learning (*d* = 0.47). These included (a) Mastery and TARGET PE models (9 studies; *d* = 0.54); (b) Interventions based on autonomy support, student choice, or Self Determination Theory (8 studies; *d* = 0.74); and (c) Practices incorporating Sport Education (7 studies; *d* = 0.67). PE intervention with three or more studies had an average effect size below the standardized mean effect for this domain were (a) fitness-based PE models (7 studies; *d* = 0.45); (b) Practices incorporating Teaching Games for Understanding (4 studies; *d* = 0.45); and (c) health-based/PA promoting interventions (8 studies; *d* = 0.11).

##### Heterogeneity of Affective Effects

The *Q*-value for these effects is 1072.120 with 70 degrees of freedom and *p* < 0.001 (*I*^2^ = 93.471%; *T*^2^ = 0.149; *T* = 0.385). The prediction interval is −0.309 to 1.245 with the true effect size for 95% of all students receiving the interventions to fall within this range.

##### To What Extent Would Publication Bias Alter These Findings?

**Figure 10:**
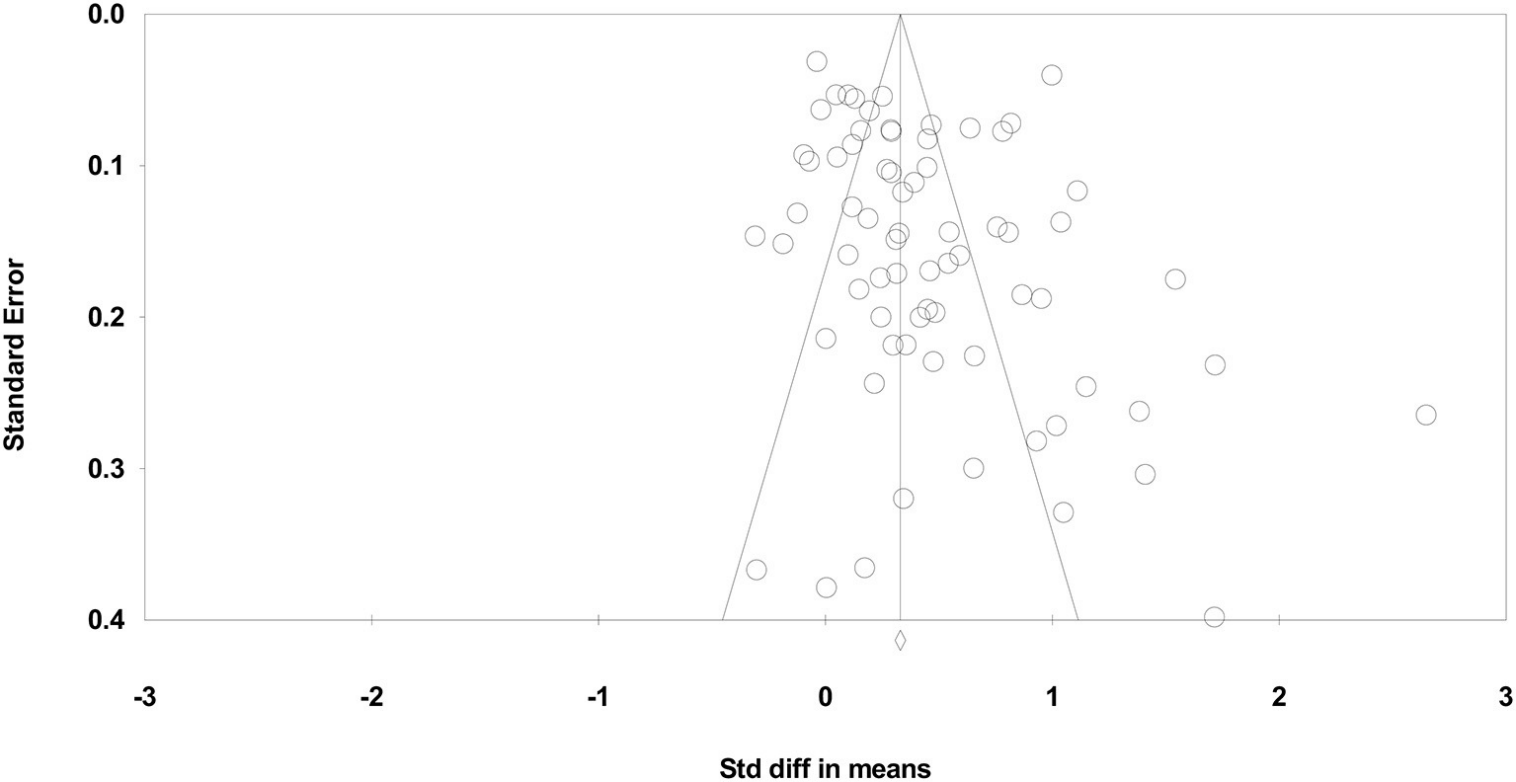
Forrest plot of standardized difference in means of affective PE interventions.

The results of the Classic fail-safe analysis showed that the incorporated data from 71 effects yielded a *z*-value of 28.241 and corresponding 2-tailed *p* < 0.0001. The fail-safe N in this case is 4671. The “Trim and Fill” analysis indicates eight studies should be trimmed from the right of the mean to reduce the potential publication bias resulting in an increased adjusted standardized effect size of *d* = 0.55 (see Figure 10).

## Discussion

The aim of the systematic review and meta-analysis was to systematically evaluate the impact of different PE lessons, instructional designs, and interventions on students' psychomotor, cognitive, affective, and social learning and development. Across the 135 studies found, there was a significant effect of PE interventions on all four domains of interest. Thus, not only can PE operate as a mechanism for enhancing learning and development broadly, specific interventions also have additive impacts over and above regular PE classes. These quantitative meta-analytic findings support the qualitative synthesis of Bailey (2006) in highlighting the multi-domain benefits of PE for development. Further, they also support claims from a range of researchers (e.g., Dudley, 2015; Cairney et al., 2019; Whitehead, 2019; Barnett et al., 2021) about the importance of the specific instructional techniques in supporting these learning and developmental outcomes to a stronger or weaker degree.

To extend our analysis of the role of PE in supporting students' learning and development, we considered the relative effect sizes between and within domains (psychomotor, cognitive, affective, and social). We note above the importance of effect sizes for comparing results across different measures, groups, and intervention designs (Glass et al., 1981; Hattie, 2009). For Hattie's (2009) analysis of the impacts related to student academic achievement, for example, the average effect equated to *d* = 0.4 (Hattie, 2009). While Hattie's (2009) analysis did not investigate PE interventions specifically, the fact that the current study finds a similar overall effect of *d* = 0.4 is important. PE interventions designed to improve student learning from a holistic multi-domain perspective are comparable to the plethora of educational studies around the world that have focused on academic achievement specifically.

An additional layer of complexity in our meta-analysis, missing from other meta-analyses of PE interventions to date, is that our data was of sufficient size and clarity to deaggregate by learning domain. Importantly, and notwithstanding our positive overall effects, we also found variation in the magnitude of specific interventions both between and within our domains of interest. Interventions for psychomotor and affective outcomes reported larger average effect sizes than for the standardized mean effect of all four domains combined (*d* = 0.40), for example, whereas cognitive and social learning interventions for cognitive and social outcomes frequently had effect sizes lower than this point.

There are two alternative explanations for this pattern of differences between domains. First, the disparities between domains may mean that QPE programs invest their time, money, and human resources in psychomotor and affective outcomes and not cognitive and social outcomes. Indeed, other subject areas and intervention sites within a broad school curriculum may be better placed to serve the cognitive and social learning needs of students. The effect sizes that we observed within each domain also appear comparable with the limited extant research on PE interventions, suggesting that these are relatively robust between-domain differences. For example, two recent reviews of PE reported medium to large positive effects on overall motor competence [i.e., *g* = 0.69 (Lorås, 2020) and ES = 0.52 (Jiménez-Díaz et al., 2019)], when compared against usual PE practice. Likewise, a recent meta-analysis of 26 studies investigating the effect of physical activity on cognitive achievement (Alvarez-Bueno et al., 2017), measured as language, mathematics, and overall performance, reported pooled effect sizes of between *d* = 0.16–0.30. Despite the robustness of our findings within domain, however, it is important to remember that effects *between* domains may not be directly comparable.

Second, and in contrast to our first explanation, the disparity in intervention effect size for psychomotor and affective development relative to cognitive and social development may simply suggest that some outcomes of interest develop more slowly than others. Observable changes in our cognitive and social outcomes of interest may emerge in smaller increments and across longer periods of time. Consistent with this explanation, we note that non-PE interventions to enhance students' social skill have similarly small, but important, effects as observed in our study: we observed an average effect for PE interventions on social learning and development of 0.32, while January et al. (2011) meta-analysis of school-wide interventions showed an average effect size on social skill of just 0.15. When considered in this light, PE interventions that target social learning and development appear particularly strong. Given our robust evidence that growth in development following PE intervention is possible across all domains, we recommend that teachers and schools seek to nurture learning and development using interventions selected above the hinge point within each domain.

Perhaps the most important findings of our meta-analysis were those showing the heterogeneity of different PE interventions on learning and development within each of the four domains. Dudley et al. (2017) argue that agencies invested in QPE, need to evaluate where their contributions to lifelong physical activity participation lie. To date, however, no research has systematically considered which PE intervention strategies are the most effective mechanisms for learning and development of school-aged youth. Our findings are therefore valuable in highlighting which intervention approaches are most successful for what developmental and learning outcomes. Within the cognitive domain, for example, the most common interventions cited as having positive impacts on cognition in the existing literature focus on increasing the amount of PE students complete (Sattelmair and Ratey, 2009) or the amount of physical activity they engage in Erickson et al. (2019). In our meta-analysis, however, we discovered that physical activity-based strategies were consistently the weakest of the PE strategies employed. Instead, games-based approaches appear to have stronger impact in this domain. This finding is consistent with recent meta-analytic research by García-Hermoso et al. (2021), who also found very small to non-significant effects for quantity-based PE interventions, and offers evidence for educators about which types of intervention have greatest cognitive benefit.

Our finding in favor of games-based approaches for cognitive development is relevant given that PE itself may take different forms. A study of the history of PE shows evolutions from gymnastics, calisthenics and fitness to sport and games in the post-war years, where it was eventually integrated with other academic pursuits (Dudley et al., 2020). The 1990's saw PE take a marked shift toward health-promotion and physical activity, spurred on by the rise of non-communicable diseases, before moving again in the past 5–7 years to a focus on UNESCO's QPE model (Dudley et al., 2020). As noted previously, however, the mechanisms for best achieving the QPE agenda for the interconnected and holistic development of youth (Dudley, 2015; Whitehead, 2019) are not well-known. While we were unable to compare the influence of the various intervention strategies in our meta-analysis on specific cognitive outcomes, such as attention, executive function, or other cognitive processing outcomes vs. conceptual learning and academic performance outcomes, our findings nonetheless highlight the need for more nuanced conversations about where our PE intervention investments should lie. If PE is to serve as a mechanism for the development of cognitive processes and cognitive learning outcomes, among other outcomes, a renewed focus on games-based pedagogies should be considered as part of QPE instruction.

There were also a few pedagogically driven PE interventions that reported effect sizes consistently above the hinge point across more than one learning domain. Whilst games-based pedagogies reported and average of *d* = 0.38 in the cognitive domain (hinge point—*d* = 0.17) it also reported an average effect of *d* = 0.58 in the psychomotor domain (hinge point—*d* = 0.52). Likewise, Mastery Teaching/TARGET interventions based on Epstein (1987) reported effects sizes of *d* = 0.94 and *d* = 0.73 and Sport Education interventions based on Siedentop (1998) reported effects sizes of *d* = 0.67 and *d* = 0.61 in the affective (hinge point—*d* = 0.47) and psychomotor learning domains, respectively. Conversely, health-based/PA promoting PE interventions reported average effect sizes below the hinge point in the cognitive (*d* = 0.06), affective (*d* = 0.11), and psychomotor (*d* = 0.13) domains.

These findings are important as the field can no longer accept that PA participation in PE alone will drive sufficient learning and development of students. Furthermore, this study vindicates and extends Hattie's (2009) contention that the art of teaching (in this case, in PE) requires deliberate intervention to ensure there is cognitive (and affective, social, and psychomotor) change in the student. The key ingredients of which are awareness of the learning and development intentions of PE and knowing enough about the pathway a student must undertake to grow and make connections in their newly acquired learning (Hattie, 2009). Future research is needed however to identify whether these strategies are consistent across years of schooling (pre, primary, and secondary) and whether there are critical points in student development where they achieve optimal effect.

Identifying the intervention strategies that the improve psychomotor, cognitive, social, and affective domains in an interconnected fashion and yield greater than average growth is a necessary step forward in actualising a QPE agenda (McLennan and Thompson, 2015).

### Limitations

While the present meta-analysis offered important insights regarding the role of specific PE interventions in supporting development within our domains of interest, there were four limitations of note. First, the study did not investigate dosage (that is, duration of interventions) for any of the intervention methods. For this reason, all comparisons should be considered a function not just of specific pedagogical approach but also potentially the way in which that program was implemented. Evidence to support the importance of dosage is mixed. While Lorås (2020), showed that duration of PE classes does not moderate the effect of specific intervention strategies on motor competence, Dudley and Burden's (2020) recent meta-analysis found a positive effect for increasing the frequency of classes. Across three learning domains (cognitive, psychomotor, and affective), with six included studies, the pooled effect-size of PE frequency on student outcomes was *d* = 0.41. While a similar focus on dosage was beyond the scope of the current study, we recommend that future research tease apart the effects of PE instructional design and PE frequency to determine their independent and combined contributions of each to student learning and development.

Second, for studies with multiple longitudinal follow-up assessments, it was also necessary to adopt a single post-intervention measurement point: in this case, the last assessment completed. We recommend that future research also consider the trajectory of change over time following promising interventions to determine how well (and for how long) their effects are maintained within each of our four domains.

Third, we did not weigh our evidence according to any formal hierarchy (e.g., for randomized controlled trials, controlled trials, or quasi-experimental studies in turn), although our analysis of methodological quality nonetheless offers opportunities to consider the strength of the reported study details when interpreting results. We recommend that future research also consider separating these designs such that conclusions about each may be compared and sampling biases understood.

Also, as in any meta-analysis, it should be noted that the instruments used to collect data for all of the four domains differed between studies. Some studies used subjective instruments and some used objective instruments to measure the same constructs, for example, while constructs themselves also varied within each domain. Moreover, as discussed above, development in some domains is inherently harder or slower to shift than in others. Finally, specific outcome measures that are common within each domain may also be more or less sensitive than others. Discretion is required to interpret our broad statistical trends in light of these conceptual and experimental differences.

## Conclusion

The rise of the UNESCO QPE agenda has important educational implications for student learning and development across four domains: cognitive, social, affective, and psychomotor. To date, however, no research has considered the impact of specific PE interventions within these domains. Such analysis is critical for determining how best to allocate the limited resources that are directed to PE globally, nationally, and locally, and for ensuring that our structural and pedagogical interventions yield the greatest potential benefits for students. Importantly, we found that almost all interventions in PE can stake a claim to making a positive difference to student learning and development. Moreover, these benefits extend across four domains: cognitive, affective, psychomotor, and social. If we continue to set the “efficacy” bar at zero, therefore, it is of little surprise that so many PE interventions claim victory in improving student outcomes. By instead considering interventions with greater than average effect within each domain, we highlight intervention approaches that are likely to enhance learning and development to the greatest extent. This is the first known study to provide clarity on where those structural and pedagogical investments lie.

## Data Availability Statement

The original contributions presented in the study are included in the article/Supplementary Material, further inquiries can be directed to the corresponding author/s.

## Author Contributions

DD, EM, and PV conceptualised the study. DD designed, performed, and analyzed all the research. PV, EM, and JC screened articles and extracted data. JC provided critical feedback and manuscript input. LB assisted in the design of the study. DD, PV, EM, and LB wrote up the research whilst also critically reviewing and editing the manuscript. All authors contributed to the article and approved the submitted version.

## Conflict of Interest

The authors declare that the research was conducted in the absence of any commercial or financial relationships that could be construed as a potential conflict of interest.

## Publisher's Note

All claims expressed in this article are solely those of the authors and do not necessarily represent those of their affiliated organizations, or those of the publisher, the editors and the reviewers. Any product that may be evaluated in this article, or claim that may be made by its manufacturer, is not guaranteed or endorsed by the publisher.
