# Supplementary material for: What Drives Quality Physical Education? A Systematic Review and Meta-Analysis of Learning and Development Effects From Physical Education-Based Interventions
Source: Front Psychol. 2022 Jun 23;13:799330. doi: 10.3389/fpsyg.2022.799330 (PMC9280720; doi:10.3389/fpsyg.2022.799330)
Supplement: Supplementary file 1 [file Data_Sheet_1.docx]

Supplementary File

References for studies included in the meta-analysis

| **Study** | **Title** | **Journal** | **Volume** | **Issue** | **Pages** | **DOI** |
| --- | --- | --- | --- | --- | --- | --- |
| Abós 2017 | Improving Students' Predisposition Towards Physical Education By Optimizing Their Motivational Processes In An Acrosport Unit | European Physical Education Review | 23 | 4 | 444-460 |  |
| Aguayo 2019 | Acute Effect Of Two Different Physical Education Classes On Memory In Children School-Age | Cognitive Development | 50 |  | 98-104 | <https://doi.org/10.1016/j.cogdev.2019.03.004> |
| Almolda-Tomas 2014 | Application Of Teaching Strategies For Improving Students' Situational Motivation In Physical Education | Electronic Journal of Research in Educational Psychology | 12 | 2 | 391-417 |  |
| Andrade 2020 | Effect Of Practice Exergames On The Mood States And Self-Esteem Of Elementary School Boys And Girls During Physical Education Classes: A Cluster-Randomized Controlled Natural Experiment | PLoS ONE | 15 | 6 |  | 10.1371/journal.pone.0232392 |
| Ardoy 2014 | A Physical Education Trial Improves Adolescents' Cognitive Performance And Academic Achievement: The Edufit Study | Scandinavian Journal of Medicine & Science in Sports | 24 | 1 | e52-e61 | https://doi.org/10.1111/sms.12093 |
| Bardaglio 2015 | Team-Teaching In Physical Education For Promoting Coordinative Motor Skills In Children: The More You Invest The More You Get | Physical Education and Sport Pedagogy | 20 | 3 | 268-282 |  |
| Barkoukis 2008 | Manipulation Of Motivational Climate In Physical Education: Effects Of A Seven-Month Intervention | European Physical Education Review | 14 | 3 | 367-387 | 10.1177/1356336x08095671 |
| Barzouka 2015 | The Effect Of Feedback Through An Expert Model Observation On Performance And Learning The Pass Skill In Volleyball And Motivation. | Journal of Physical Education & Sport | 15 | 3 | 407-416 |  |
| Bechter 2019 | A Cluster-Randomized Controlled Trial To Improve Student Experiences In Physical Education: Results Of A Student-Centered Learning Intervention With High School Teachers | Psychology of Sport and Exercise | 45 |  |  | <https://doi.org/10.1016/j.psychsport.2019.101553> |
| Benítez-Sillero 2021 | Intervention Programme To Prevent Bullying In Adolescents In Physical Education Classes (Prebullpe): A Quasi-Experimental Study. | Physical Education & Sport Pedagogy | 26 | 1 | 36-50 |  |
| Bortoli 2015 | The Effects Of Motivational Climate Interventions On Psychobiosocial States In High School Physical Education | Research Quarterly for Exercise and Sport | 86 | 2 | 196-204 |  |
| Boržíková 2020 | Development Of Basic Motor Competencies Among Standard Population During The Prepubertal Period | Journal of Physical Education & Sport | 20 | 6 | 3699-3705 |  |
| Boyle-Holmes 2010 | Promoting Elementary Physical Education: Results Of A School-Based Evaluation Study | Health Education & Behavior | 37 | 3 | 377-389 | 10.1177/1090198109343895 |
| Breslin 2012 | The Effect Of Teachers Trained In A Fundamental Movement Skills Programme On Children'S Self-Perceptions And Motor Competence | European Physical Education Review | 18 | 1 | 114-126 |  |
| Browne 2004 | A Comparison Of Rugby Seasons Presented In Traditional And Sport Education Formats | European Physical Education Review | 10 | 2 | 199-214 | 10.1177/1356336x04044071 |
| Carlson 2008 | Physical Education And Academic Achievement In Elementary School: Data From The Early Childhood Longitudinal Study | American Journal of Public Health | 98 | 4 | 721-727 | 10.2105/AJPH.2007.117176 |
| Cecchini 2007 | Effects Of Personal And Social Responsibility On Fair Play In Sports And Self-Control In School-Aged Youths | European Journal of Sport Science | 7 | 4 | 203-211 | 10.1080/17461390701718497 |
| Cecchini 2020 | Effect Of A Target-Based Intervention On Students? Motivational Change: A Study Throughout An Academic Year In Physical Education | Journal of Teaching in Physical Education | 39 | 2 | 186-195 | 10.1123/jtpe.2018-0357 |
| Chatoupis 2017 | Effects Of Two Practice Style Formats On Fifth Grade Students' Motor Skill Performance And Task Engagement | Physical Educator | 74 | 2 |  |  |
| Chatzipanteli 2015 | Self-Regulation, Motivation And Teaching Styles In Physical Education Classes: An Intervention Study | Journal of Teaching in Physical Education | 34 | 2 | 333-344 | 10.1123/jtpe.2013-0024 |
| Chen 2008 | Content Specificity Of Expectancy Beliefs And Task Values In Elementary Physical Education. | Research Quarterly for Exercise & Sport | 79 | 2 | 195-208 |  |
| Cheon 2019 | An Intervention To Help Teachers Establish A Prosocial Peer Climate In Physical Education | Learning and Instruction | 64 |  | 101223 | <https://doi.org/10.1016/j.learninstruc.2019.101223> |
| Coe 2006 | Effect Of Physical Education And Activity Levels On Academic Achievement In Children | Med Sci Sports Exerc | 38 | 8 | 1515-9 | 10.1249/01.mss.0000227537.13175.1b |
| Cohen 2012 | The Effectiveness Of Aligned Developmental Feedback On The Overhand Throw In Third-Grade Students | Physical Education and Sport Pedagogy | 17 | 5 | 525-541 |  |
| Coimbra 2021 | Impact Of A Physical Education-Based Behavioural Skill Training Program On Cognitive Antecedents And Exercise And Sport Behaviour Among Adolescents: A Cluster-Randomized Controlled Trial. | Physical Education & Sport Pedagogy | 26 | 1 | 16-35 |  |
| Colella 2019 | Teaching Styles, Physical Literacy And Perceived Physical Self-Efficacy. Results Of A Learning Unit In Primary School. | Spor Hekimligi Dergisi/Turkish Journal of Sports Medicine | 54 |  | 1-7 |  |
| Cöster 2018 | Extended Physical Education In Children Aged 6–15 Years Was Associated With Improved Academic Achievement In Boys | Acta Paediatrica | 107 | 6 | 1083-1087 | 10.1111/apa.14278 |
| Costigan 2016 | High-Intensity Interval Training For Cognitive And Mental Health In Adolescents | Medicine & Science in Sports & Exercise | 48 | 10 | 1985-93 | 10.1249/mss.0000000000000993 |
| Cuevas 2016 | Sport Education Model And Self-Determination Theory: An Intervention In Secondary School Children | Kinesiology | 48 | 1 | 30-38 |  |
| Dalziell 2015 | Better Movers And Thinkers (Bmt): An Exploratory Study Of An Innovative Approach To Physical Education | Europe's journal of psychology | 11 | 4 | 722 |  |
| Dalziell 2019 | Better Movers And Thinkers: An Evaluation Of How A Novel Approach To Teaching Physical Education Can Impact Children'S Physical Activity, Coordination And Cognition | British Educational Research Journal | 45 | 3 | 576-591 |  |
| Debruijn 2020 | Effects Of Aerobic And Cognitively-Engaging Physical Activity On Academic Skills: A Cluster Randomized Controlled Trial. | Journal of Sports Sciences | 38 | 15 | 1806-1817 |  |
| Digelidis 2003 | A One-Year Intervention In 7Th Grade Physical Education Classes Aiming To Change Motivational Climate And Attitudes Towards Exercise. | Psychology of Sport & Exercise | 4 | 3 | 195-210 |  |
| Duncan 2019 | Sequencing Effects Of Object Control And Locomotor Skill During Integrated Neuromuscular Training In 6- To 7-Year-Old Children | Journal of Strength & Conditioning Research | 33 | 8 | 2262-2274 |  |
| Eather 2016 | Effects Of Exercise On Mental Health Outcomes In Adolescents: Findings From The Crossfit™ Teens Randomized Controlled Trial | Psychology of Sport and Exercise | 26 |  | 14-23 | 10.1016/j.psychsport.2016.05.008 |
| Ellis 1995 | Effectiveness Of A Collaborative Consultation Approach To Basic Concept Instruction With Kindergarten Children | Language, Speech, and Hearing Services in Schools | 26 | 1 | 69-74 | 10.1044/0161-1461.2601.69 |
| Ericsson 2008 | Motor Skills, Attention And Academic Achievements. An Intervention Study In School Years 1-3 | British Educational Research Journal | 34 | 3 | 301-313 |  |
| Ericsson 2014 | Motor Skills And School Performance In Children With Daily Physical Education In School—A 9‐Year Intervention Study | Scandinavian Journal of Medicine & Science in Sports | 24 | 2 | 273-278 | 10.1111/j.1600-0838.2012.01458.x |
| Escartí 2010 | Implementation Of The Personal And Social Responsibility Model To Improve Self-Efficacy During Physical Education Classes For Primary School Children | International Journal of Psychology & Psychological Therapy | 10 | 3 | 387-402 |  |
| Felver 2020 | School-Based Yoga Intervention Increases Adolescent Resilience: A Pilot Trial | Journal of Child and Adolescent Mental Health |  |  |  | 10.2989/17280583.2019.1698429 |
| Fernandez-Rio 2017 | Impact Of A Sustained Cooperative Learning Intervention On Student Motivation | Physical Education and Sport Pedagogy | 22 | 1 | 89-105 | 10.1080/17408989.2015.1123238 |
| Fisher 2011 | Effects Of A Physical Education Intervention On Cognitive Function In Young Children: Randomized Controlled Pilot Study | BMC Pediatrics | 11 | 1 | 97 | 10.1186/1471-2431-11-97 |
| Font-Lladó 2020 | A Pedagogical Approach To Integrative Neuromuscular Training To Improve Motor Competence In Children: A Randomized Controlled Trial. | Journal of Strength & Conditioning Research | 34 | 11 | 3078-3085 |  |
| Franco 2017 | The Effects Of A Physical Education Intervention To Support The Satisfaction Of Basic Psychological Needs On The Motivation And Intentions To Be Physically Active | Journal of Human Kinetics | 59 | 1 | 5-15 | doi:10.1515/hukin-2017-0143 |
| Fu 2016 | Effect Of The Spark Program On Physical Activity, Cardiorespiratory Endurance, And Motivation In Middle-School Students | Journal of Physical Activity & Health | 13 | 5 | 534-542 | 10.1123/jpah.2015-0351 |
| García-Calvo 2016 | Effects Of An Intervention Programme With Teachers On The Development Of Positive Behaviours In Spanish Physical Education Classes | Physical Education and Sport Pedagogy | 21 | 6 | 572-588 |  |
| Gibbons 1995 | Fair Play For Kids: Effects On The Moral Development Of Children In Physical Education. | Research Quarterly for Exercise & Sport | 66 | 3 | 247-255 |  |
| Gibbons 2010 | The Impact Of An Experiential Education Program On The Self-Perceptions And Perceived Social Regard Of Physical Education Students | Journal of Sport & Exercise Psychology | 32 | 6 | 786-804 |  |
| Gil-Arias 2017 | Impact Of A Hybrid Tgfu-Sport Education Unit On Student Motivation In Physical Education | PLoS ONE | 12 | 6 |  | 10.1371/journal.pone.0179876 |
| Gråstén 2017 | Effects Of School-Based Physical Activity Program On Students' Moderate-To-Vigorous Physical Activity And Perceptions Of Physical Competence. | Journal of Physical Activity & Health | 14 | 6 | 455-464 |  |
| Grasten 2019 | Social Competence And Moderate To Vigorous Physical Activity Of School-Aged Children Through A Creative Physical Education Intervention | Advances in Physical Education |  |  | 1-16 |  |
| Gray 2011 | Developing Pupils’ Performance In Team Invasion Games | Physical Education and Sport Pedagogy | 16 | 1 | 15-32 | 10.1080/17408980903535792 |
| Greco 2020 | An Extracurricular Physical Education Intervention Improves The Body Image Self-Perception In Adolescents | European Journal of Fitness, Nutrition and Sport Medicine Studies | 1 | 1 |  |  |
| Gu 2018 | Impact Of A Pedometer-Based Goal-Setting Intervention On Children'S Motivation, Motor Competence, And Physical Activity In Physical Education | Physical Education and Sport Pedagogy | 23 | 1 | 54-65 |  |
| Hagins 2016 | Yoga Improves Academic Performance In Urban High School Students Compared To Physical Education: A Randomized Controlled Trial | Mind, Brain, and Education | 10 | 2 | 105-116 | 10.1111/mbe.12107 |
| Hartmann 2010 | Effects Of A School-Based Physical Activity Program On Physical And Psychosocial Quality Of Life In Elementary School Children: A Cluster-Randomized Trial. | Pediatric Exercise Science | 22 | 4 | 511-522 |  |
| Harvey 2017 | Middle And Elementary School Students’ Changes In Self-Determined Motivation In A Basketball Unit Taught Using The Tactical Games Model | Journal of Human Kinetics | 59 |  | 39 |  |
| Hernández 2020 | Effect Of Autonomy Support And Dialogic Learning On School Children’S Physical Activity And Sport | Scandinavian Journal of Psychology |  |  |  | 10.1111/sjop.12637 |
| Hortz 2008 | Social Cognitive Theory Variables Mediation Of Moderate Exercise | American Journal of Health Behavior | 32 | 3 | 305-314 | 10.5993/AJHB.32.3.8 |
| How 2013 | The Effects Of Choice On Autonomous Motivation, Perceived Autonomy Support, And Physical Activity Levels In High School Physical Education. | Journal of Teaching in Physical Education | 32 | 2 | 131-148 |  |
| Ignico 2006 | The Effects Of An Intervention Strategy On Children'S Heart Rates And Skill Performance | Early Child Development and Care | 176 | 7 | 753-761 | 10.1080/03004430500232615 |
| Ilker 2013 | The Effects Of Different Motivational Climates On Students' Achievement Goals, Motivational Strategies And Attitudes Toward Physical Education | Educational Psychology | 33 | 1 | 59-74 | 10.1080/01443410.2012.707613 |
| Jaakkola 2006 | Changes In Students’ Self‐Determined Motivation And Goal Orientation As A Result Of Motivational Climate Intervention Within High School Physical Education Classes | International Journal of Sport and Exercise Psychology | 4 | 3 | 302-324 | 10.1080/1612197X.2006.9671800 |
| Jamner 2004 | A Controlled Evaluation Of A School-Based Intervention To Promote Physical Activity Among Sedentary Adolescent Females: Project Fab | Journal of Adolescent Health | 34 | 4 | 279-289 | <https://doi.org/10.1016/j.jadohealth.2003.06.003> |
| Jansen 2018 | Increased Physical Education At School Improves The Visual-Spatial Cognition During Adolescence | Educational Psychology | 38 | 7 | 964-976 |  |
| Jarani 2016 | Effects Of Two Physical Education Programmes On Health- And Skill-Related Physical Fitness Of Albanian Children | Journal of Sports Sciences | 34 | 1 | 35-46 | 10.1080/02640414.2015.1031161 |
| Kalaja 2012 | Development Of Junior High School Students' Fundamental Movement Skills And Physical Activity In A Naturalistic Physical Education Setting | Physical Education and Sport Pedagogy | 17 | 4 | 411-428 |  |
| Karabourniotis 2002 | Curriculum Enrichment With Self-Testing Activities In Development Of Fundamental Movement Skills Of First-Grade Children In Greece | Perceptual and Motor Skills | 94 | 3_suppl | 1259-1270 | 10.2466/pms.2002.94.3c.1259 |
| Kliziene 2018 | Effects Of A 7-Month Exercise Intervention Programme On The Psychosocial Adjustment And Decrease Of Anxiety Among Adolescents | European Journal of Contemporary Education | 7 | 1 | 127-136 |  |
| Kokkonen 2019 | Effectiveness Of A Creative Physical Education Intervention On Elementary School Students' Leisure-Time Physical Activity Motivation And Overall Physical Activity In Finland. | European Physical Education Review | 25 | 3 | 796-815 |  |
| Kouli 2009 | The Effects Of An Aerobic Program On Health-Related Fitness And Intrinsic Motivation In Elementary School Pupils | Studies in Physical Culture & Tourism | 16 | 3 | 301-306 |  |
| Kriellaars 2019 | The Impact Of Circus Arts Instruction In Physical Education On The Physical Literacy Of Children In Grades 4 And 5 | Journal of Teaching in Physical Education | 38 | 2 | 162-170 |  |
| Krüger 2018 | Second Language Acquisition Effects Of A Primary Physical Education Intervention: A Pilot Study With Young Refugees | PLoS ONE | 13 | 9 |  | 10.1371/journal.pone.0203664 |
| Lakes 2004 | Promoting Self-Regulation Through School-Based Martial Arts Training | Journal of Applied Developmental Psychology | 25 | 3 | 283-302 |  |
| Lander 2017 | Improving Early Adolescent Girls' Motor Skill: A Cluster Randomized Controlled Trial | Medicine & Science in Sports & Exercise | 49 | 12 | 2498-2505 | 10.1249/mss.0000000000001382 |
| Leptokaridou 2014 | Experimental Longitudinal Test Of The Influence Of Autonomy-Supportive Teaching On Motivation For Participation In Elementary School Physical Education | Educational Psychology | 36 | 7 | 1138-1159 | 10.1080/01443410.2014.950195 |
| Lima 2020 | Effects Of A Physical Education Intervention On Academic Performance: A Cluster Randomised Controlled Trial | International Journal of Environmental Research & Public Health | 17 | 12 |  | 10.3390/ijerph17124287 |
| lisahunter 2014 | Active Kids Active Minds: A Physical Activity Intervention To Promote Learning? | Asia-Pacific Journal of Health, Sport and Physical Education | 5 | 2 | 117-131 |  |
| Lonsdale 2019 | An Internet-Supported School Physical Activity Intervention In Low Socioeconomic Status Communities: Results From The Activity And Motivation In Physical Education (Amped) Cluster Randomised Controlled Trial | British Journal of Sports Medicine | 53 | 6 | 341-347 | 10.1136/bjsports-2017-097904 |
| Lopes 2017 | Effectiveness Of Physical Education To Promote Motor Competence In Primary School Children | Physical Education and Sport Pedagogy | 22 | 6 | 589-602 |  |
| Lubans 2018 | School Physical Activity Intervention Effect On Adolescents' Performance In Mathematics | Medicine and Science in Sports and Exercise | 50 | 12 | 2442-2450 |  |
| Marshall 1997 | The Effects Of Quality Daily Physical Education On Movement Competency In Obese Versus Nonobese Children | Adapted Physical Activity Quarterly | 14 | 3 | 222-237 |  |
| Martin 2009 | Motivational Climate And Fundamental Motor Skill Performance In A Naturalistic Physical Education Setting. | Physical Education & Sport Pedagogy | 14 | 3 | 227-240 |  |
| Martínez-López 2018 | 24 Sessions Of Monitored Cooperative High-Intensity Interval Training Improves Attention-Concentration And Mathematical Calculation In Secondary School | Work | 46 |  | 25 |  |
| Mathisen 2016 | Effects Of School-Based Intervention Program On Motor Performance Skills. | Journal of Physical Education & Sport | 16 | 3 | 737-742 |  |
| Mayorga-Vega 2012 | Effect Of A Physical Fitness Program On Physical Self-Concept And Physical Fitness Elements In Primary School Students. | Perceptual & Motor Skills | 115 | 3 | 984-996 |  |
| McKenzie 1998 | Effects Of A Physical Education Program On Children'S Manipulative Skills | Journal of Teaching in Physical Education | 17 | 3 | 327-341 |  |
| Meijer 2020 | The Effects Of Aerobic Versus Cognitively Demanding Exercise Interventions On Executive Functioning In School-Aged Children: A Cluster-Randomized Controlled Trial | Journal of Sport & Exercise Psychology | 43 | 1 | 1-13 | 10.1123/jsep.2020-0034 |
| Miller 2016 | Can Physical Education And Physical Activity Outcomes Be Developed Simultaneously Using A Game-Centered Approach? | European Physical Education Review | 22 | 1 | 113-133 |  |
| Moreno-Murcia 2019 | Effect Of A Teaching Intervention On Motivation, Enjoyment, And Importance Given To Physical Education | Motricidade | 15 | 2-3 | 21-31 |  |
| Morgan 2002 | Effects Of Manipulating The Motivational Climate In Physical Education Lessons | European Physical Education Review | 8 | 3 | 207-229 | 10.1177/1356336X020083003 |
| Neumark-Sztainer 2010 | New Moves—Preventing Weight-Related Problems In Adolescent Girls: A Group-Randomized Study | American Journal of Preventive Medicine | 39 | 5 | 421-432 | https://doi.org/10.1016/j.amepre.2010.07.017 |
| Neville 2021 | Effect Of A Six-Week Dance-Based Physical Education Intervention On Primary School Children'S Creativity: A Pilot Study. | European Physical Education Review | 27 | 1 | 203-220 |  |
| Noggle 2012 | Benefits Of Yoga For Psychosocial Well-Being In A Us High School Curriculum: A Preliminary Randomized Controlled Trial | Journal of Developmental and Behavioral Pediatrics | 33 | 3 | 193-201 | 10.1097/DBP.0b013e31824afdc4 |
| O'Brien 2008 | The Effects Of A Body-Focused Physical And Health Education Module On Self-Objectification And Social Physique Anxiety In Irish Girls | Journal of Teaching in Physical Education | 27 | 1 | 116-126 | 10.1123/jtpe.27.1.116 |
| Osterlie 2018 | Can Flipped Learning Enhance Adolescents’ Motivation In Physical Education? An Intervention Study | Journal for Research in Arts and Sports Education | 2 | 1 |  | 10.23865/jased.v2.916 |
| Pagona 2008 | The Development Of Motor Creativity In Elementary School Children And Its Retention | Creativity Research Journal | 20 | 1 | 72-80 | 10.1080/10400410701842078 |
| Palmer 2018 | A Physical Education Intervention Effects On Correlates Of Physical Activity And Motivation | Health Promot Pract | 19 | 3 | 455-464 | 10.1177/1524839917707740 |
| Pardo 2016 | Motivational Outcomes And Predictors Of Moderate-To-Vigorous Physical Activity And Sedentary Time For Adolescents In The Sigue La Huella Intervention | International Journal of Behavioral Medicine | 23 | 2 | 135-142 | 10.1007/s12529-015-9528-5 |
| Perlman 2010 | Change In Affect And Needs Satisfaction For Amotivated Students Within The Sport Education Model | Journal of Teaching in Physical Education | 29 | 4 | 433-445 | 10.1123/jtpe.29.4.433 |
| Pesce 2012 | Benefits Of Multi-Sports Physical Education In The Elementary School Context | Health Education Journal | 72 | 3 | 326-336 |  |
| Pesce 2016 | Deliberate Play And Preparation Jointly Benefit Motor And Cognitive Development: Mediated And Moderated Effects | Frontiers in Psychology | 7 | 349 |  | 10.3389/fpsyg.2016.00349 |
| Pesce 2021 | Fostering Self-Control Development With A Designed Intervention In Physical Education: A Two-Year Class-Randomized Trial | Child Development | 92 | 3 | 937-958 | https://doi.org/10.1111/cdev.13445 |
| Pietsch 2017 | Cognitive Motor Coordination Training Improves Mental Rotation Performance In Primary School-Aged Children | Mind, Brain, and Education | 11 | 4 | 176-180 |  |
| Platvoet 2016 | Four Weeks Of Goal-Directed Learning In Primary Physical Education Classes | Perceptual and Motor Skills | 122 | 3 | 871-885 | 10.1177/0031512516648729 |
| Polvi 2000 | The Use Of Cooperative Learning As A Social Enhancer In Physical Education | Scandinavian Journal of Educational Research | 44 | 1 | 105-115 | 10.1080/713696660 |
| Potdevin 2018 | How Can Video Feedback Be Used In Physical Education To Support Novice Learning In Gymnastics? Effects On Motor Learning, Self-Assessment And Motivation | Physical Education and Sport Pedagogy | 23 | 6 | 559-574 |  |
| Prusak 2004 | The Effects Of Choice On The Motivation Of Adolescent Girls In Physical Education. | Journal of Teaching in Physical Education | 23 | 1 | 19-29 |  |
| Reed 2013 | Examining The Impact Of 45 Minutes Of Daily Physical Education On Cognitive Ability, Fitness Performance, And Body Composition Of African American Youth | Journal of Physical Activity & Health | 10 | 2 | 185-197 | 10.1123/jpah.10.2.185 |
| Robertson 2018 | Savouring Our Mistakes: Learning From The Fitquest Project | International Journal of Child-Computer Interaction | 16 |  | 55-67 | 10.1016/j.ijcci.2017.12.003 |
| Rubeli 2020 | Promoting Schoolchildren'S Self-Esteem In Physical Education: Testing The Effectiveness Of A Five-Month Teacher Training | Physical Education and Sport Pedagogy | 25 | 4 | 346-360 |  |
| Sallis 1999 | Effects Of Health-Related Physical Education On Academic Achievement: Project Spark | Research Quarterly for Exercise and Sport | 70 | 2 | 127-134 | 10.1080/02701367.1999.10608030 |
| Sánchez-Oliva 2017 | Effects Of An Intervention With Teachers In The Physical Education Context: A Self-Determination Theory Approach | PLoS ONE | 12 | 12 |  | 10.1371/journal.pone.0189986 |
| Schmidt 2013 | Promoting A Functional Physical Self-Concept In Physical Education: Evaluation Of A 10-Week Intervention | European Physical Education Review | 19 | 2 | 232-255 | 10.1177/1356336x13486057 |
| Schmidt 2015 | Delayed Positive Effects Of An Acute Bout Of Coordinative Exercise On Children’s Attention | Perceptual and Motor Skills | 121 | 2 | 431-446 | 10.2466/22.06.PMS.121c22x1 |
| Schmidt 2015a | Cognitively Engaging Chronic Physical Activity, But Not Aerobic Exercise, Affects Executive Functions In Primary School Children: A Group-Randomized Controlled Trial | Journal of Sport & Exercise Psychology | 37 | 6 | 575-591 | 10.1123/jsep.2015-0069 |
| Schnider 2021 | Effects Of Behavioural Skill Training On Cognitive Antecedents And Exercise And Sport Behaviour In High School Students: A Cluster-Randomised Controlled Trial | International Journal of Sport and Exercise Psychology |  |  |  | 10.1080/1612197X.2021.1877329 |
| Sgrò 2020 | Assessing The Effect Of Different Teaching Strategies On Students' Affective Learning Outcomes During Volleyball Lessons | Journal of Physical Education & Sport | 20 |  | 2136-2142 |  |
| Sharpe 1995 | The Effects Of A Sportsmanship Curriculum Intervention On Generalized Positive Social Behavior Of Urban Elementary School Students | Journal of Applied Behavior Analysis | 28 | 4 | 401-416 | https://doi.org/10.1901/jaba.1995.28-401 |
| Sparks 2017 | An Intervention To Improve Teachers? Interpersonally Involving Instructional Practices In High School Physical Education: Implications For Student Relatedness Support And In-Class Experiences | Journal of Sport and Exercise Psychology | 39 | 2 | 120-133 | 10.1123/jsep.2016-0198 |
| Spittle 2009 | The Influence Of Sport Education On Student Motivation In Physical Education | Physical Education and Sport Pedagogy | 14 | 3 | 253-266 | 10.1080/17408980801995239 |
| Stojadinović 2020 | Physical Education And Music Education In The Context Of The Possibility Of Using The Integrated Learning Approach As A Reflection Of Modern Society. | Facta Universitatis: Series Physical Education & Sport | 18 | 3 | 621-634 |  |
| Sun 2012 | Learning Science-Based Fitness Knowledge In Constructivist Physical Education | The Elementary School Journal | 113 | 2 | 215-229 | 10.1086/667405 |
| Telford 2012 | Physical Education, Obesity, And Academic Achievement: A 2-Year Longitudinal Investigation Of Australian Elementary School Children | American Journal of Public Health | 102 | 2 | 368-374 | 10.2105/ajph.2011.300220 |
| van Beurden 2003 | Can We Skill And Activate Children Through Primary School Physical Education Lessons? 'Move It Groove It'--A Collaborative Health Promotion Intervention | Preventive Medicine: An International Journal Devoted to Practice and Theory | 36 | 4 | 493-501 | 10.1016/S0091-7435(02)00044-0 |
| van der Fels 2020 | Effects Of Aerobic Exercise And Cognitively Engaging Exercise On Cardiorespiratory Fitness And Motor Skills In Primary School Children: A Cluster Randomized Controlled Trial. | Journal of Sports Sciences | 38 | 17 | 1975-1983 |  |
| Velez 2010 | The Impact Of A 12-Week Resistance Training Program On Strength, Body Composition, And Self-Concept Of Hispanic Adolescents | Journal of Strength and Conditioning Research | 24 | 4 | 1065-1073 |  |
| Viciana 2020 | A Sport Education Teaching Unit As A Citizenship Education Strategy In Physical Education. A Group-Randomized Controlled Trial. | Retos: Nuevas Perspectivas de Educación Física, Deporte y Recreación |  | 38 | 44-52 |  |
| Wallhead 2004 | Effects Of A Sport Education Intervention On Students’ Motivational Responses In Physical Education | Journal of Teaching in Physical Education | 23 | 1 | 4-18 |  |
| Wallhead 2014 | Effect Of A Sport Education Program On Motivation For Physical Education And Leisure-Time Physical Activity | Research Quarterly for Exercise and Sport | 85 | 4 | 478-487 |  |
| Weiss 2015 | Effectiveness Of A School-Based Fitness Program On Youths' Physical And Psychosocial Health Outcomes. | Pediatric Exercise Science | 27 | 4 | 547-557 |  |
| Yli-Piipari 2018 | Motivational Pathways To Leisure-Time Physical Activity Participation In Urban Physical Education: A Cluster-Randomized Trial | Journal of Teaching in Physical Education | 37 | 2 | 123-132 | 10.1123/jtpe.2017-0099 |
| You 2013 | Influence Of A Health-Related Physical Fitness Model On Students' Physical Activity, Perceived Competence, And Enjoyment. | Perceptual & Motor Skills | 117 | 3 | 956-970 |  |
| Zhu 2016 | Physical Activity And Situational Interest In Mobile Technology Integrated Physical Education: A Preliminary Study. | Acta Gymnica | 46 | 2 | 59-67 |  |
| Zourbanos 2013 | The Effects Of A Self-Talk Intervention On Elementary Students’ Motor Task Performance | Early Child Development and Care | 183 | 7 | 924-930 | 10.1080/03004430.2012.693487 |

Supplementary File

PRISMA 2020 Checklist

| **Section and Topic** | **Item #** | **Checklist item** | **Location where item is reported** |
| --- | --- | --- | --- |
| **TITLE** | | |  |
| Title | 1 | Identify the report as a systematic review. | Title page |
| **ABSTRACT** | | |  |
| Abstract | 2 | See the PRISMA 2020 for Abstracts checklist. | Abstract |
| **INTRODUCTION** | | |  |
| Rationale | 3 | Describe the rationale for the review in the context of existing knowledge. | Pg. 3-4 |
| Objectives | 4 | Provide an explicit statement of the objective(s) or question(s) the review addresses. | Pg. 5 |
| **METHODS** | | |  |
| Eligibility criteria | 5 | Specify the inclusion and exclusion criteria for the review and how studies were grouped for the syntheses. | Pg. 6 |
| Information sources | 6 | Specify all databases, registers, websites, organisations, reference lists and other sources searched or consulted to identify studies. Specify the date when each source was last searched or consulted. | Pg. 7 |
| Search strategy | 7 | Present the full search strategies for all databases, registers and websites, including any filters and limits used. | Pg. 7 |
| Selection process | 8 | Specify the methods used to decide whether a study met the inclusion criteria of the review, including how many reviewers screened each record and each report retrieved, whether they worked independently, and if applicable, details of automation tools used in the process. | Pg. 8 |
| Data collection process | 9 | Specify the methods used to collect data from reports, including how many reviewers collected data from each report, whether they worked independently, any processes for obtaining or confirming data from study investigators, and if applicable, details of automation tools used in the process. | Pg. 8 |
| Data items | 10a | List and define all outcomes for which data were sought. Specify whether all results that were compatible with each outcome domain in each study were sought (e.g. for all measures, time points, analyses), and if not, the methods used to decide which results to collect. | Pg. 8 |
|  | 10b | List and define all other variables for which data were sought (e.g. participant and intervention characteristics, funding sources). Describe any assumptions made about any missing or unclear information. | Pg. 8 |
| Study risk of bias assessment | 11 | Specify the methods used to assess risk of bias in the included studies, including details of the tool(s) used, how many reviewers assessed each study and whether they worked independently, and if applicable, details of automation tools used in the process. | Pg. 8-9 |
| Effect measures | 12 | Specify for each outcome the effect measure(s) (e.g. risk ratio, mean difference) used in the synthesis or presentation of results. | Pg. 9 |
| Synthesis methods | 13a | Describe the processes used to decide which studies were eligible for each synthesis (e.g. tabulating the study intervention characteristics and comparing against the planned groups for each synthesis (item #5)). | Pg. 7 |
|  | 13b | Describe any methods required to prepare the data for presentation or synthesis, such as handling of missing summary statistics, or data conversions. | Pg. 7-8 |
|  | 13c | Describe any methods used to tabulate or visually display results of individual studies and syntheses. | N/A |
|  | 13d | Describe any methods used to synthesize results and provide a rationale for the choice(s). If meta-analysis was performed, describe the model(s), method(s) to identify the presence and extent of statistical heterogeneity, and software package(s) used. | Pg. 9 |
|  | 13e | Describe any methods used to explore possible causes of heterogeneity among study results (e.g. subgroup analysis, meta-regression). | Pg. 9-10 |
|  | 13f | Describe any sensitivity analyses conducted to assess robustness of the synthesized results. | N/A |
| Reporting bias assessment | 14 | Describe any methods used to assess risk of bias due to missing results in a synthesis (arising from reporting biases). | Pg. 8 |
| Certainty assessment | 15 | Describe any methods used to assess certainty (or confidence) in the body of evidence for an outcome. | N/A |
| **RESULTS** | | |  |
| Study selection | 16a | Describe the results of the search and selection process, from the number of records identified in the search to the number of studies included in the review, ideally using a flow diagram. | Pg. 10  Figure 1 |
|  | 16b | Cite studies that might appear to meet the inclusion criteria, but which were excluded, and explain why they were excluded. | Figure 1 (citations not provided) |
| Study characteristics | 17 | Cite each included study and present its characteristics. | Table 1 |
| Risk of bias in studies | 18 | Present assessments of risk of bias for each included study. | Table 3 |
| Results of individual studies | 19 | For all outcomes, present, for each study: (a) summary statistics for each group (where appropriate) and (b) an effect estimate and its precision (e.g. confidence/credible interval), ideally using structured tables or plots. | Figures 3,5,7,9 |
| Results of syntheses | 20a | For each synthesis, briefly summarise the characteristics and risk of bias among contributing studies. | Pg. 12-20 |
|  | 20b | Present results of all statistical syntheses conducted. If meta-analysis was done, present for each the summary estimate and its precision (e.g. confidence/credible interval) and measures of statistical heterogeneity. If comparing groups, describe the direction of the effect. | Pg. 12-20 |
|  | 20c | Present results of all investigations of possible causes of heterogeneity among study results. | N/A |
|  | 20d | Present results of all sensitivity analyses conducted to assess the robustness of the synthesized results. | N/A |
| Reporting biases | 21 | Present assessments of risk of bias due to missing results (arising from reporting biases) for each synthesis assessed. | Pg. 12-20 |
| Certainty of evidence | 22 | Present assessments of certainty (or confidence) in the body of evidence for each outcome assessed. | N/A |
| **DISCUSSION** | | |  |
| Discussion | 23a | Provide a general interpretation of the results in the context of other evidence. | Pg. 20-22 |
|  | 23b | Discuss any limitations of the evidence included in the review. | Pg. 24-25 |
|  | 23c | Discuss any limitations of the review processes used. | Pg. 24 |
|  | 23d | Discuss implications of the results for practice, policy, and future research. | Pg. 22, 25 |
| **OTHER INFORMATION** | | |  |
| Registration and protocol | 24a | Provide registration information for the review, including register name and registration number, or state that the review was not registered. | Pg. 6 |
|  | 24b | Indicate where the review protocol can be accessed, or state that a protocol was not prepared. | Pg. 6 |
|  | 24c | Describe and explain any amendments to information provided at registration or in the protocol. | N/A |
| Support | 25 | Describe sources of financial or non-financial support for the review, and the role of the funders or sponsors in the review. | Front matter |
| Competing interests | 26 | Declare any competing interests of review authors. | Front matter |
| Availability of data, code and other materials | 27 | Report which of the following are publicly available and where they can be found: template data collection forms; data extracted from included studies; data used for all analyses; analytic code; any other materials used in the review. | Front matter |
